# Clays and the Origin of Life: The Experiments

**DOI:** 10.3390/life12020259

**Published:** 2022-02-09

**Authors:** Jacob Teunis (Theo) Kloprogge, Hyman Hartman

**Affiliations:** 1 School of Earth and Environmental Sciences, The University of Queensland, Brisbane, QLD 4072, Australia; 2 Department of Chemistry, College of Arts and Sciences, University of the Philippines Visayas, Miagao 5023, Philippines; 3 Department of Earth Atmospheric and Planetary Sciences, Massachusetts Institute of Technology, 77 Massachusetts Avenue, Cambridge, MA 02139, USA

**Keywords:** clay, catalysis, Mars, Earth, montmorillonite, nontronite, saponite, organic reactions, origin of life, smectite

## Abstract

There are three groups of scientists dominating the search for the origin of life: the organic chemists (the Soup), the molecular biologists (RNA world), and the inorganic chemists (metabolism and transient-state metal ions), all of which have experimental adjuncts. It is time for Clays and the Origin of Life to have its experimental adjunct. The clay data coming from Mars and carbonaceous chondrites have necessitated a review of the role that clays played in the origin of life on Earth. The data from Mars have suggested that Fe-clays such as nontronite, ferrous saponites, and several other clays were formed on early Mars when it had sufficient water. This raised the question of the possible role that these clays may have played in the origin of life on Mars. This has put clays front and center in the studies on the origin of life not only on Mars but also here on Earth. One of the major questions is: What was the catalytic role of Fe-clays in the origin and development of metabolism here on Earth? First, there is the recent finding of a chiral amino acid (isovaline) that formed on the surface of a clay mineral on several carbonaceous chondrites. This points to the formation of amino acids on the surface of clay minerals on carbonaceous chondrites from simpler molecules, e.g., CO_2_, NH_3_, and HCN. Additionally, there is the catalytic role of small organic molecules, such as dicarboxylic acids and amino acids found on carbonaceous chondrites, in the formation of Fe-clays themselves. Amino acids and nucleotides adsorb on clay surfaces on Earth and subsequently polymerize. All of these observations and more must be subjected to strict experimental analysis. This review provides an overview of what has happened and is now happening in the experimental clay world related to the origin of life. The emphasis is on smectite-group clay minerals, such as montmorillonite and nontronite.

## 1. Introduction

One of the most intriguing questions in science has been the origin of life on Earth. Several hypotheses have been proposed to explain this. Since the time of Darwin’s “warm little pond”, a large number of different theories have been published [1]. Currently, there is still a large gap in our understanding of how life on Earth was formed. The early history of Earth, roughly between 4.5 and 4.1 billion years ago, is still largely shrouded in mystery, since there are no rocks from this era that we can use to reconstruct the early environments in which life was formed and evolved. The very first fossils that we know of from about 3.5 billion years ago already show amazing complexity. How did this life come to be?

One of the first experimental approaches to tackle the formation of the earliest organic molecules was the Miller–Urey experiment (also known as the Miller experiment) in 1952. It simulated the conditions believed at the time to exist on Earth in its earliest stages of life and assessed the potential chemical origin of life. It used simple chemical compounds, such as water (H_2_O), methane (CH_4_), ammonia (NH_3_), and hydrogen (H_2_). These compounds were then placed inside a sealed sterilized glass flask, to which a second smaller flask half-filled with water was attached. The water in the second flask was heated, and the water vapor formed was free to move to the first flask. Uninterrupted electrical sparking between two electrodes in the first flask was used to replicate lightning in the water vapor and gaseous mix. Subsequently, the atmospheric temperature was lowered, allowing the water to condense and drip into a U-shaped trap underneath the system. After 24 h, the condensed liquid accumulated in the trap had a pink color, while after 7 days of uninterrupted running, the condensed liquid had turned deep red in color and had become cloudy [2]. After the reaction was stopped, paper chromatography allowed the identification of five different amino acids: glycine, α-alanine, and β-alanine were detected, whereas the presence of aspartic acid and α-aminobutyric acid was not determined with absolute certainty because the spots were hard to observe. After Stanley Miller’s passing in 2007, the preserved sealed samples from his original tests were reanalyzed, and it was found that there were in fact more than 20 different amino acids formed. This was significantly more than the number detected by Stanley Miller and more than the 20 amino acids that can be found in the genetic code in nature. More recent results indicated that the initial atmosphere on Earth may have had a different composition compared to the gas mixture employed by Miller in his tests, though prebiotic trials still show the formation of racemic mixtures of simple-to-complex molecules, e.g., cyanide, when applying a number of different conditions [3].

At the time, Miller’s experiment seemed to support Alexander Oparin’s [4] as well as J. B. S. Haldane’s [5] theory postulating that the environments on early Earth aided chemical reactions that resulted in the formation of more complex organic molecules from simpler inorganic precursors. Oparin summarized a route that he believed could explain the production of microscopic localized systems from basic organic chemicals, now generally known as the droplet theory, from which primitive living things could have developed [4]. He thought that the order of different steps in the origin of life could be as follows: first, the formation of cells, followed by enzymes, and finally, genes. This was based on the observation that when an appropriate oily liquid is combined with water, these two different liquids occasionally create a stable mixture known as a coacervate, where the oily liquid is dispersed as small droplets that remain in suspension in the water. Coacervate droplets can be produced simply through nonbiological processes. These droplets show some superficial resemblance to living cells.

Manfred Eigen offered a different theory, which was basically the Oparin theory in reverse [6,7]. In his theory, genes came first, followed by enzymes and finally cells. It was widely accepted at the time for two reasons. To start with, Eigen and Orgel [8] conducted experiments employing RNA as the starting material and thought it plausible that RNA replication formed the basic process and that the rest of biology developed around it. The discovery of the double helix structure revealed that genes have simpler structures compared to enzymes. Now that the secret of the genetic code was finally understood, it was no more than natural to assume that nucleic acids were formed as primary compounds and that proteins were formed as secondary compounds. Eigen’s hypothesis started with self-replicating RNA, followed by the appearance of enzymes a little later, creating a primitive form of the current genetic transcription mechanism with RNA, and finally, cells appeared to provide it all with the necessary physical cohesion. To date, there has been an enormous amount of experimentation to test these theories, and none has brought us any closer to understanding the origin of life on Earth.

**Figure 1:**
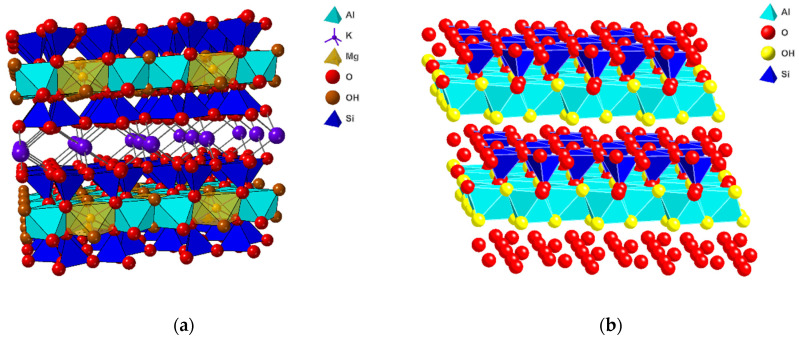
Two examples of typical crystal structures of common clay minerals: (**a**) montmorillonite, a 2:1 type smectite with layers consisting of two tetrahedral sheets and one octahedral sheet with layers separated by the interlayer space with hydrated cations; (**b**) kaolinite, a 1:1 type clay mineral with layers consisting of one tetrahedral and one octahedral sheet.

In our view, the most promising theory to explain the origin of life is centered around the interaction of active sites on clay mineral surfaces with simple organic molecules. This idea was first introduced by Cairns-Smith in 1966 [9]. He proposed that during the formation of a crystal of a mineral, particular types of lattice defects (e.g., dislocations) usually replicate as a necessary part of the crystallization process. Since those imperfections seem to replicate themselves, they are thus self-selecting, so any crystallization process is likely to involve a rudimentary biological evolution. In 1975, Hartman [10] used this idea to suggest that metabolism could have developed from a simple environment instead of a complex one. There was no mention of metabolism in the 1966 paper by Cairns-Smith [9]. Clays are able to replicate and drive the evolution of metabolism; they have the catalytic ability to synthesize monomers (amino acids, nucleotides, etc.) and polymerize them, resulting in RNA–peptide worlds in which RNA replicates (genes) and, in cooperation with coded peptides, drives the evolution of the cell. There is a large variety of clay minerals, but from the perspective of this paper, the group of smectites is the most important (Figure 1). This group consists of clay minerals with a layer structure, where each layer contains a central octahedral sheet located in between two tetrahedral sheets. In the tetrahedral sheets, Si^4+^ is the most important element, but substitution by Al^3+^ and, to a lesser extent, Fe^3+^ creates a negative charge. The composition of the octahedral sheet is much more varied with cation (e.g., Mg^2+^, Fe^2+^, Fe^3+^, Al^3+^, and Li^+^) vacancies, and depending on the composition, it can add an additional negative charge to the layer or partially compensate for the negative charge of the tetrahedral sheets. Since the layers have an overall net negative charge, they are separated by the interlayer space, where hydrated interlayer cations, such as Na^+^, K^+^, and Ca^2+^, compensate for the negative layer charge. As a result, these smectites have a number of important properties that are of interest for developing the theory on the origin of life, such as cation-exchange capacity, swellability, presence of acid sides, etc.

## 2. Clay Minerals on Early Earth

There are very few rocks still present on the Earth’s surface from its early days. Pre-early Archean (before 3.5 billion years ago) crust is thought to have been predominantly composed of magmatic rocks in the form of basalt and komatiite lavas [11]. During the Hadean and early Archean, the geothermal gradient was probably much higher than it is now, and convection cells in the mantle were much smaller and more rapid [12]. Hence, the surface of early Earth most likely consisted of solidified lava containing cooled solid blocks comprising peridotite (olivine-rich, high Fe and Mg content)-derived rocks. Consequently, the komatiite–basalt crust was recycled faster than the current ocean floor. Hence, despite the possible presence of some granitoids older than 4 billion years, the Earth’s crust during that period consisted primarily of komatiitic–basalts that created the ocean floor and emerging plateaus.

The original chemical composition of these peridotite and komatiite rocks favored the formation of Fe-Mg clay minerals (saponite, hectorite, and nontronite) instead of Al-rich clay minerals (montmorillonite and beidellite) (Table 1). Fe-Mg clay minerals were produced within chemical microsystems due to seawater weathering or hydrothermal alteration, mostly by post-magmatic processes. Under current atmospheric conditions, magmatic minerals (olivine and pyroxene, which are generally high in Mg and Fe) and their subsequent metamorphic reaction products (serpentine) will readily react when in contact with meteoritic water, and they are then initially altered to trioctahedral phyllosilicates such as talc, kerolite, or stevensite–saponite. When weathering increases in intensity, these secondary minerals will become destabilized, resulting in the formation of dioctahedral Fe-rich clay minerals (mainly nontronite). Because of the lack of oxygen in the Hadean and early Archean atmosphere, iron ions would be mostly in the ferrous state. However, nontronite was discovered in significant quantities on the surface of Mars (see Section 3).

During the Hadean, oceans were much larger than they are now and covered most of the Earth’s surface [13]. Consequently, seawater weathering of the cooled and brecciated margins of basalt and komatiite pillow lavas or flows most likely drove major clay-forming processes. Although seawater had a different composition compared to current seawater as well as different conditions (e.g., increased temperatures, slightly more acidic (pH 5–6), and dissolved iron concentration close to 100 ppm) [14], it is likely that the mineral reactions were not that different from current reactions because the reactions were buffered by the chemical/mineralogical composition of the rock. Arndt et al. [15,16] indicated that komatiite lava surfaces were generally vitreous and brecciated. Interactions with seawater throughout the quenching stage possibly resulted in the formation of a palagonite-like crust (mixture of altered glass with embryos of clay minerals) in which Fe-Mg clay minerals were formed. During the Hadean and early Archean, this type of weathering at low temperature did not take place for periods long enough to allow the formation of clay minerals in similar amounts compared to other alteration or post-magmatic processes. In fact, seawater was repeatedly heated to boiling as a result of large meteorite impacts and rapidly heated rocks while it circulated through fracture networks produced by the impact shocks [17].

## 3. Clay Minerals Found in Our Solar System

The occurrence of clay minerals is not restricted to our own planet but are likewise present on the Martian surface and in/on meteorites, asteroids, and comets.

### 3.1. Mars

The discovery and characterization of clay minerals on Mars have mainly been based on orbital very-near-IR (VNIR) spectra between approximately 0.4 and 5 mm. Thermal infrared (TIR) spectra obtained in the mid-IR (MIR) range between approximately 200 and 2000 cm^−1^ (approximately 5–50 mm) have similarly provided orbital data about the mineralogy of Mars, including clay minerals. The Mars Science Laboratory (MSL) rover launched by NASA in 2011, landing on Mars in 2012, employs an XRD instrument known as CheMin (short for “Chemistry and Mineralogy”) to detect clay minerals present on the Martian surface at its landing site within the Gale Crater [18], and a number of different Martian missions have obtained surface chemistry information, which was then utilized for the deduction of the possible mineralogical composition [19,20,21]. The most important data for clay minerals on Mars were provided by the OMEGA [22] and CRISM [23] orbital imaging spectrometers, which detected sunlight reflected back from the surface of Mars in the VNIR spectral range. The Fe/Mg-smectites on Mars are mostly detected in rocks about 4 billion years old. Viking’s elemental analyses of surface material led to the hypothesis that clay minerals probably existed on the surface of Mars. Modeling of the major element results seemed to be in agreement with the presence of about 60–80 wt.% smectite [19,20]. It was not until 2004, when the Observatoire pour la Minéralogie, l’Eau, les Glaces et l’Activité (OMEGA) VNIR imaging spectrometer on Mars Express began observing the surface of Mars at approximately 1–3 km resolution, that clay minerals were conclusively detected [24]. Once the Compact Reconnaissance Imaging Spectrometer for Mars (CRISM) instrument on the Mars Reconnaissance Orbiter started measuring targeted high-resolution (18 m/pixel) hyperspectral imagery in 2006, areas with various sized clay mineral-containing outcrops were found almost everywhere where ancient rocks were exposed on the Martian surface [25,26].

The initial evaluation of clay minerals observed on Mars using OMEGA data suggesting that the clay minerals occurred mostly in the old Noachian terrains seems to be correct for most of the planet’s surface [27]. Younger clay-rich deposits do exist in some isolated areas, such as Noctis Labyrinthus, e.g., [28], Coprates Chasma, e.g., [29], and some impact craters. Initial studies of clay minerals containing outcrops in Mawrth Vallis showed that Al-rich clay minerals were consistently found in strata younger than those with Fe/Mg-rich smectite [30]. A later investigation determined that this is true for most of the surface of Mars where clay minerals are exposed [31]. Identification of the clay minerals through analytical instruments onboard the Curiosity rover in the Gale crater provided evidence for the formation of smectites in Hesperian rocks in environments characterized by a low water-to-rock ratio (see, e.g., [32,33,34,35]). Beyond the preserved diversity of clay minerals (smectites comprising nontronite, saponite, beidellite and montmorillonite, vermiculites, corrensites, chlorites, K/Al-rich micas, mixed-layer Fe^2+^-smectite/mica, kaolinite, mixed-layered kaolinite–smectite, allophane, talc, and serpentines), planetwide mineralogical trends occur. Most of Mars’ alteration (more than 80% of the deposits) seems to have resulted in the formation of Fe/Mg-rich clay minerals with a high smectitic contribution [36,37,38]. The domination of Fe/Mg-rich clay minerals over other clay minerals on Mars is in agreement with water-restricted alteration of the original basaltic and ultramafic rocks, though the related geological environment and associated alteration reactions continue to be a mystery in some instances. A major part of the alteration reactions probably took place in the Martian subsurface, although it is evident that surface alteration reactions did take place all over ancient Mars.

### 3.2. Meteorites

A meteorite is defined as a solid piece of debris from an object, such as a comet, asteroid, or meteoroid, originating from space and surviving the fall through the atmosphere to impact the surface of a planet or moon. As such, meteorites deliver extraterrestrial clay minerals from space to Earth. Meteorites are classified based on their petrology and origin, with certain meteorites originating from the Moon or Mars, while numerous other meteorites originate from the asteroid belt, found mainly in between the orbits of Jupiter and Mars, or other origins. Most meteorites are of the stony type (chondrites and achondrites), whereas a smaller number belong to the iron or stony-iron types [39,40,41]. Lunar and Martian meteorites belong to the achondrite category.

Early observations of clay minerals and other altered matter present in carbonaceous chondrites have been published for the Allende [42], Plainview [43], and Mokoia [44] meteorites, although whether these clay minerals were formed by terrestrial alteration after arriving on the Earth’s surface or were of extraterrestrial origin was a matter of intense discussion among scientists. Carbonaceous chondrites belonging to the Ivuna and Orgueil types have clay minerals that are thoroughly blended with carbon-rich matter, interpreted as a preterrestrial alteration through reactions with aqueous solutions [45]. The C2 Tagish Lake meteorite contains a combination of clay minerals, carbon, carbonate minerals, and sulfide minerals that are likewise believed to be of preterrestrial origin because of the very brief contact with the Earth’s environment of this meteorite [46]. Most of the Martian meteorites fall into one of three categories: shergottites, nakhlites, and chassignites, with the names derived from the locations where these meteorites were found [47]. The question of whether the clay minerals observed in these particular meteorites were produced through terrestrial weathering instead of preterrestrial reactions has been a matter of intense debate between scientists for many years and was only commonly recognized after large amounts of clay minerals were found on the surface of Mars. Ongoing studies of clay minerals in these meteorites resulted in the identification of mainly Fe-rich smectite [48]. Regrettably, the clay minerals found in meteorites are generally present in very small amounts, on the scale of nanometers or smaller, therefore making identification and characterization extremely difficult. The nakhlite meteorite group has the highest concentration of clay minerals, and therefore, these meteorites have been and are being studied much more thoroughly than others. The most common clay minerals observed consist of high-Fe^3+^/^2+^-saponite as well as serpentine [48]. Modeling of the geochemical environments necessary for the crystallization of the detected carbonate minerals and Fe/Mg-smectite in nakhlite-type meteorites suggests that there had to be hydrothermal fluid rich in CO_2_ at a temperature between 150 and 200 °C with a pH of approximately 6–8 and a water/rock ratio of approximately 300 [49].

### 3.3. Asteroids and Comets

Information about the hydrated mineral composition of asteroids is essential for understanding the origin of water on Earth, understanding the meteorite record, and solving problems related to the processes that occurred in the beginning of the history of our solar system. Jones et al. [50] studied a series of low-albedo objects, mainly belonging to the C class and its subclasses (using the Tholen asteroid taxonomy [51]) and concluded that the percentage of hydrated asteroids declined with the increase in the semimajor axis from the center of the asteroid belt in an outward direction. They thought that the asteroids initially consisted of a blend of ice together with anhydrous silicates and that the hydrated minerals were produced by aqueous reactions instead of in the initial nebula. In their opinion, in the middle of the belt, hydrated asteroids’ temperatures were sufficiently high to melt the internal ice and produce phyllosilicates, whereas at no time did the outer-belt asteroids reach temperatures sufficiently high to melt ice. The C, B, G, and F asteroids were suggested to encompass an alteration series ([52] and references therein). These four subclasses each have various amounts of hydrated minerals, ranging from the common hydrated G asteroids to the nearly anhydrous F asteroids. This suggests an alteration series, from the G asteroids, with temperature increases to where melted ice could result in widespread aqueous alteration, to the F asteroids, with temperature increases so high that hydrated minerals broke down [53].

The asteroid Ceres (classified as a dwarf planet since 2006), detected for the first time on 1 January 1801 by Giuseppe Piazzi using the Palermo Astronomical Observatory in Sicily (Italy), is the biggest object in the asteroid belt (940 km diameter) and consists mainly of silicate minerals [54,55]. Spectral features in the near 3 mm telescopic data first raised the idea that clay minerals existed on Ceres [56]. The presence of ammonium-containing clay minerals was based on telescopic measurements of spectral bands around 3.07 mm [57,58]. These data were most consistent with NH_4_-saponite. Further investigation of better telescopic data of Ceres additionally showed the existence of carbonate as well as Fe-smectite [59]. A relatively recent review by Berg et al. [60] of telescopic spectral data of asteroids and NH_4_-containing minerals indicated that, besides Ceres, 10-Hygiea and 324-Bamberga also showed spectral characteristics around 3.05–3.07 mm, attributed to ammonium-clay minerals. A number of additional spectral bands are typically observed for NH_4_-exchanged clay minerals, though these particular bands are more challenging to observe in telescopic spectra of asteroids. Consequently, the present-day interpretation of the Ceres data validates the existence of ammonium-containing clay minerals, although the exact nature of the clay minerals remains to be identified.

The Spitzer Space Telescope has measured TIR spectra (5–40 mm) of a number of Jupiter-family comets, among which is 9B-Tempel 1 [61]. The Spitzer Telescope spectra were measurements of dust particles ejected from the coma of 9B-Tempel 1 prior to and directly following the impact of the Deep Impact mission (launched by NASA in 2005) on its nucleus on 4 July 2005. Spectral features related to clay minerals, carbonate minerals, water ice, and organic compounds were observed in the Spitzer spectral data of the dust particles directly following the impact [62]. Fine particles of crystalline as well as amorphous olivine and pyroxenes were detected in the dust particles around 9B-Tempel 1 prior to and immediately following the impact and have been modeled in combination with the clay minerals, carbonate minerals, water ice, and organic compounds [63]. Lisse et al. [62] determined that nontronite corresponded best to the clay mineral spectral features and estimated a concentration of approximately 5–10% in the dust particles. Next, Lisse et al. [64] assessed the Spitzer Telescope spectral data for comet 9B-Tempel 1 relative to the International Space Observatory (ISO) spectral data of comet Hale–Bopp after its 1995–1996 apparition caused significant dust outflow [65] and an ISO spectrum of HD100546, a young star frequently used for comparison to comets [66]. Spectral analyses of these three bodies suggested the presence of similar components, including nontronite, for comet Hale–Bopp [64].

## 4. The Search for Life on Mars

History was made in 1976 with NASA’s Viking Project after it became the very first U.S. spacecraft to land successfully on the Martian surface and produced the first photos of its surface that were successfully sent back to Earth. Two indistinguishable spacecraft, both comprising a lander together with an orbiter, were constructed. Both orbiter–lander pairs were launched together and successfully entered Mars orbit; the landers later separated from the orbiters and landed on the surface of Mars. The Viking 1 lander landed on the western slope of Chryse Planitia (the Plains of Gold), whereas the Viking 2 lander landed at Utopia Planitia. In addition to obtaining images and accumulating other scientific information on the surface of Mars, each lander performed several identical biological experiments created to search for potential signs of life.

In the labeled release (LR) experiment, a soil sample from the surface of Mars was injected with a small volume consisting of a highly diluted aqueous nutrient solution. The nutrients used in the experiment (seven molecules that were found as products in the original Miller–Urey experiment, see Section 1) were labeled with radioactive ^14^C. The atmosphere over the soil was examined for the release of radioactive ^14^CO_2_ (and/or additional carbon-based) gas, which would be proof of microorganisms living in the Martian soil metabolizing one or more of the injected nutrient compounds [67]. This was then followed by the control experiment, as explained for the pyrolytic release (PR) experiment in the next paragraph. The outcome was literally unexpected, given the negative results of the gas chromatograph–mass spectrometer (GCMS) analyses, which showed no significant volumes of organic molecules in the Martian soil (actually, less carbon was found than in the soils collected during the Apollo landings on the Moon), with a steady evolution of radioactive gases from the soil directly after the initial injection. The same test was performed by the two Viking landers: the first lander took a soil sample from the surface exposed to sunlight, while the second lander used a soil sample from underneath a rock not exposed to sunlight; in both instances, the first injections yielded positive results [68]. Next, sterilization control experiments were performed by heating several soil samples. Soil samples treated for 3 h at 160 °C produced no radioactive gas after the nutrients had been injected, while soil samples treated for 3 h at only 50 °C showed a significant decrease in evolved radioactive gas after the nutrient injection [69]. A soil sample kept at a temperature of 10 °C for a number of months exhibited substantially lower radioactive gas release [70]. Miller et al. [71] speculated that the observed delays in the experiment’s chemical reactions suggested the presence of biological activity comparable to the circadian rhythm detected in terrestrial cyanobacteria. In 2012, Bianciardi et al. [72] suggested the discovery of “extant microbial life on Mars” on the basis of mathematical speculation using cluster analysis of the original labeled release tests onboard the 1976 Viking Mission.

The pyrolytic release (PR) experiment involved the usage of light and water, together with a carbon-containing atmosphere containing both carbon monoxide (CO) and carbon dioxide (CO_2_), replicating the Martian atmosphere. The carbon-bearing gases were labeled with the radioactive isotope ^14^C. The idea was that if there were photosynthetic organisms present, these organisms would take up a certain amount of the carbon as biomass via carbon fixation, similar to plants and cyanobacteria on Earth. After an incubation period of a selected number of days, the atmospheric gases were eliminated from the system, the remaining soil was then heated at 650 °C, and the products released were collected in an instrument that measured radioactivity. If some part of the original ^14^C from the experimental atmosphere had been changed into biomass, it would be released in the gas phase while the soil was being heated, and the radioactivity counter could then measure it as proof of the presence of life. In the case that a positive result was detected, a second sample of the same soil would be heated in order to “sterilize” it. Next, the “sterilized” soil would be examined as a control, and should it again produce the same result, then that was to be interpreted as proof that the observed activity was chemical in nature and not biological. However, a null or greatly diminished result would be proof of biological activity. This same control “sterilized” soil sample approach was employed for all other life detection experiments (GCMS, gas exchange (GEX), and LR) if any of them provided an initial positive result [73]. The first evaluation of data produced by the Viking 1 PR tests suggested that

“analysis of the results shows that a small but significant formation of organic matter occurred” and that the sterilized control showed no evidence of organics, showing that the “findings could be attributed to biological activity [74]”.

However, based on the persistence of the release of organic compounds at 90 °C, the inhibition of organic compounds after the injection of water vapor, and, in particular, the lack of organic molecules detected in the soil from Mars by the GCMS test, it was determined that a nonbiological origin of the PR data was the best explanation [73,75]. Nevertheless, in later years, when the GCMS results were increasingly questioned, the PR data were once more perceived as potentially consistent with a biological origin, even if

“an explanation for the apparent small synthesis of organic matter in the pyrolytic release experiment remains obscure [76]”.

Organic molecules are believed to be widespread in our solar system, e.g., on asteroids, meteorites, comets, and other icy bodies orbiting the Sun, so observing no evidence of organic compounds on the Martian surface was unexpected. The GCMS was functioning as expected, since the controls were successful, and it could measure traces of chlorine, probably originating from the cleaning solvents employed to sterilize the instruments before the launch [77]. A review in 2018 of the original GCMS data indicates that certain organic molecules may in fact have been measured, supporting the results from the Curiosity rover [25]. Back then, the complete lack of organic compounds on the surface of Mars cast doubt on the outcome of the biological tests, because metabolism requiring organic molecules was what the experiment was supposed to measure. These days, it is presumed that the original Viking’s biological experiments are inconclusive and can be described entirely by chemical reactions [68,74,78]. In August 2008, the Phoenix lander observed perchlorate, a strongly oxidizing molecule at temperatures over 200 °C, which was first believed to represent a false-positive LR effect [79]. Nevertheless, the results of tests run by Navarro-Gonzáles et al. [80] may be an indication that organic molecules “could have been present” in the soil tested by Viking 1 and Viking 2, because in 2008, the Phoenix lander measured perchlorate, which is able to break down organic molecules when heated, forming chloromethane together with dichloromethane as a secondary product, the same chlorine molecules found by each Viking lander after they completed identical tests on Mars. Since perchlorate could have resulted in the breakdown of any Martian organic molecules present, the question of whether or not Viking found organic molecules is still a matter of debate, as other chemical and biological explanations are possible [74,81]. In addition, it has been claimed that the labeled release (LR) test observed such a small number of metabolizing organisms in the soil from the Martian surface that it would have made it very difficult for the GCMS to measure them [68]. This is believed by the LR experiment’s designer, Gilbert Levin, who still regards the positive LR outcomes as an indication of the presence of life on Mars [82]. He and other scientists have been trying to replicate the original Viking results with either biological or nonbiological compounds on Earth. Though not a single test has yet exactly reproduced the Mars LR experiment and control data, tests using hydrogen peroxide (H_2_O_2_)-saturated titanium dioxide (TiO_2_) have generated some comparable outcomes [83]. Although most astrobiologists now assume that the Viking biological tests were inconclusive at best or negative, Gilbert Levin is not the only scientist to think otherwise. The present argument for the existence of life on Mars is based on older data reinterpreted based on new advances in science [84,85]. In 2006, Navarro-González et al. [85] showed that the Viking biological experiments probably did not have the sensitivity to detect trace amounts of organic molecules. In 2010, Navarro-González et al. [80] indicated that if organic molecules were present in the Martian soil, they could not have been measured since heating the soil to check for the presence of organic molecules would cause the perchlorate to break them down quickly, forming chloromethane and dichloromethane, as the Viking landers detected. In addition, they stated that this is not evidence for the presence of life, though it could influence what scientists will use in the future to search for organic biosignatures.

The search for life on Mars will almost certainly not end until upcoming missions to Mars either convincingly show the existence of life on Mars, identify the chemical compound(s) explaining the Viking data, or both. Since the initial Viking landings, a number of different landers and rovers have successfully been deployed on the Martian surface. Mars Pathfinder was a NASA spacecraft that put a base station with the first roving probe on the surface of Mars back in 1997. It comprised a lander, rechristened the Carl Sagan Memorial Station, together with a lightweight wheeled robotic Mars rover called Sojourner, the very first rover functioning on another body in our solar system besides the Earth–Moon system. The mission contained a set of analytical instruments to study the atmosphere, climate, and geology of Mars, including the chemical composition of its rocks and soil on the surface, but did not specifically look for proof of life [86].

Spirit, also called MER-A (Mars Exploration Rover-A or MER-2), was another rover on the surface of Mars, working for just over 6 years from 2004 to 2010. It was the first of two identical rovers as part of NASA’s Mars Exploration Rover Mission. Spirit touched down without any problems within the impact crater Gusev on Mars on 4 January 2004, while its twin, Opportunity (MER-B), landed three weeks later on the other side of the planet. Its main mission was to search for and analyze different minerals, rocks, and soils that could possess indications about past water activity. Of special interest were minerals formed by water-related interactions, e.g., precipitation, evaporation, sedimentary cementation, and hydrothermal activity. NASA’s search for proof of life on Mars started with the initial question of whether the environment on Mars was, at any time in its history, appropriate for the development of life. All life forms on Earth need water, and therefore, studying the historical record of water on the Martian surface is of vital importance. While the Mars Exploration Rovers could not observe life directly, they provided very valuable data on the habitability of the Martian environment throughout the geological history of Mars [87,88].

Phoenix was a space probe that touched down on the surface of Mars on 25 May 2008 and functioned only for a short period (until 2 November 2008). Its analytical instruments were employed to evaluate the regional habitability and to study the geological history of water on the surface of Mars. Its mission had two main objectives. The first objective was to obtain more information about the geological history of water, as it is essential to understanding climate changes over time. The other objective was to assess historical or prospective current planetary habitability at the ice–soil interface. Its analytical instruments were appropriate for obtaining data associated with the geological and potentially biological history of the Arctic region on Mars. Phoenix was the very first lander to provide results from one of the poles, which aided NASA’s key approach for Mars exploration: “Follow the water” [89]. The Mars Science Laboratory mission succeeded in landing the Curiosity rover on 6 August 2012. Its objectives comprise a study of the climate and geology on Mars and determining whether Mars could have supported life at any time, involving the examination of the role of water and planetwide habitability. The most important instrument for the search for life is the Chemistry and Camera complex (ChemCam), which contains two remote sensing instruments brought together in a single setup: laser-induced breakdown spectroscopy (LIBS) and a remote micro-imager (RMI) telescope [90].

The Interior Exploration employing Seismic Investigations, Geodesy and Heat Transport (InSight) mission consists of a lander investigating the deep interior of Mars. It successfully landed at Elysium Planitia on Mars in late 2018. Perseverance is a Mars rover the size of a car and was developed to study the crater Jezero as a component of its mission. Its successful landing on Mars was confirmed on 18 February 2021 [91]. The Perseverance rover has four main science objectives that include, among others, searching for habitability, identifying past environments that may have been able to support microbial life and searching for biosignatures, and looking for evidence of possible past microbial life in those habitable environments, especially in particular rock types known to preserve evidence throughout history [92]. Very recently, China also managed to successfully put a lander and rover on the surface of Mars. Tianwen-1 (TW-1; Heavenly Questions) arrived in Martian orbit on 10 February 2021. During the first three months, it examined possible landing sites from its reconnaissance orbit. On 14 May 2021, the lander/rover combination successfully touched down on the surface of Mars, and thus, China became the third country to both land successfully on and communicate with a lander/rover on the surface of Mars, together with the former Soviet Union and the United States [93].

Astrobiological exploration of Mars continued with the ExoMars Trace Gas Orbiter in 2016. The first part consisted of a mission launched in 2016, placing the Trace Gas Orbiter (TGO) in orbit around Mars in October 2016, followed by releasing the Schiaparelli EDM lander. The orbiter is functioning well; unfortunately, the lander crashed on the surface of Mars. The second component of this mission was intended to be launched in July 2020, with the Kazachok lander delivering the Rosalind Franklin rover to the surface of Mars, establishing a scientific mission that was anticipated to run well into 2022 or later. On 12 March 2020, it was revealed that this component had been postponed to 2022 due to complications with the parachutes that could not be solved in the short timeframe before the launch window. Currently, the revised plan is that in June 2023, the lander known as Kazachok (which means “little Cossack” in Russian, in addition to referring to a folk dance) will put the Rosalind Franklin rover onto the surface of Mars. The TGO is, besides a Mars telecommunications orbiter serving to maintain contact with its controllers on Earth, an atmospheric gas analyzer mission. It maps the origin of methane release as well as other gases on Mars and, thus, will support the selection of the ultimate landing site for the Rosalind Franklin rover, now expected to be launched sometime in 2022. The existence of methane in the Martian atmosphere is interesting since the probable cause is either release from currently present life or from geological activity. After the Rosalind Franklin rover lands sometime in 2023, the TGO will be relocated in a lower orbit, where it can execute analytical research in addition to acting as a telecommunication relay for the Rosalind Franklin rover. The Rosalind Franklin rover is expected to land on the surface of Mars in June 2023, after which it is intended to travel autonomously across the surface of Mars. The onboard instrumentation will comprise the exobiology laboratory suite, called the “Pasteur analytical laboratory”, which will search for the existence of biomolecules and biosignatures from past life. The Pasteur setup will comprise the Mars Organic Molecule Analyzer (MOMA), MicrOmega-IR, and the Raman Laser Spectrometer (RLS) [94].

For the near future, several programs have been proposed to further the understanding of the geology of Mars and the potential for life on its surface. Icebreaker Life is one of the Mars lander mission ideas recommended for NASA’s Discovery Program [95]. It comprises a stationary lander that will consist of almost a clone of the successful Phoenix and InSight spacecraft; however, in contrast, it will contain astrobiology instruments, comprising a drill for collecting ice–soil samples in the northern plains in order to look for biosignatures related to present-day or past life near the surface of Mars [96]. The payload will include the Signs Of Life Detector (SOLID), which will be able to detect complete cells, particular complex organic compounds, and polymers using fluorescence immunoassays [97]. Employing one Life-Detection Chip (LDCHIP) assessing a few square centimeters [98], SOLID’s antibody library will be able to recognize up to 300 different organic molecules. In total, the instrument is expected to contain 16 Life-Detection Chips. Furthermore, the laser desorption mass spectrometer (LDMS) will be able to measure and characterize numerous nonvolatile organic molecules. The LDMS employs a pulsed laser desorption/ionization (LDI) method, where ions can be detected directly from particulate matter under Martian atmospheric pressure, with no need for vacuum loading. The major advantage of the LDMS is that it is not affected by the presence of perchlorate.

The Biological Oxidant and Life Detection (BOLD) has been presented as a concept mission to Mars and is centered around the search for proof or biosignatures of microscopic life on Mars. The major aim of BOLD is to measure the concentration of hydrogen peroxide (H_2_O_2_) present in the soil on the Martian surface, as well as to seek processes characteristic of life. Six landing packages, expected to touch down on the surface of Mars, consist of a restricted power supply, together with a series of oxidant and life-detection experiments. The Fluorescent Stain (FS) experiment will entail two different tests, both based on biochemical principles observed for organisms on Earth. The first experiment is based on the assumption that DNA will be present in potential organisms on Mars, while the second is based on the existence of esterases. Although photochemically formed oxidants may in part cause the oxidation of compounds in the soil on Mars, a potential photochemical source for oxidants in aqueous solutions appears to be more difficult to explain. The broad belief is that the most probable oxidant molecule is formed by hydrogen peroxide (H_2_O_2_), generated by photochemical reactions in the Martian atmosphere, and subsequently disperses into the soil [99,100]. An identical origin is thought to be responsible for the oxidant species in older aqueous environments [101]. However, H_2_O_2_ is an unstable molecule under the environmental conditions found close to the Martian surface, such as in its acidic environments [102]. Under such circumstances, the short lifetime of H_2_O_2_ makes it very challenging to measure, but certain potential techniques do exist. A possible method uses the leuco crystal violet (LCV) technique of Mottola et al. [103]. This process was recently enhanced for H_2_O_2_ measurement to determine concentrations in the micromolar to a few hundred nanomolar range in Fe-containing solutions with various pH values [104]. The LCV process comprises 4,4′,4″-methylidynetris oxidation with H_2_O_2_ and horseradish peroxidase, producing the crystal violet ion, CV^+^, which absorbs light at 590 nm. The colored CV^+^ is stable for several days, making the technique independent of the direct use of a spectrophotometer and, consequently, particularly suitable for in situ analyses on Mars. Almost every biologically produced chiral molecule is formed as one or the other enantiomer. For instance, every amino acid present in living organisms on Earth consists of left-handed chiral molecules, whereas every sugar in nucleic acids is a right-handed molecule. Therefore, a few of the same labeled compounds (e.g., alanine and lactate) together with certain additional chiral molecules will be incorporated into the BOLD mission. Should labeled gas be released solely or primarily from a soil sample injected with one of the isomers, in contrast to its enantiomer, this would form clear proof of a biological reaction. No use or the utilization of both stereoisomers would point to a lack of biological reactions. This test could be performed in a comparable fashion to the initial labeled release experiment on the Viking mission or in a revised form [105].

## 5. Nontronite and Related Iron-Rich Smectites

The initial onset of life with the simplest organic molecules on planets such as Mars may be contingent on the existence of water at the surface. It is not essential that this water was/is present in the form of liquid or crystalline water, but it may very well exist as interlayer water, as is present in smectitic clays. A particular group of smectites important for the search for the origin of planetary life consists of smectites that have high iron content, generally known as ferruginous smectites and nontronites [27,31,106,107,108]. On Earth, clay minerals can be found in weathering layers and soils, continental and marine sediments, volcanic deposits, geothermal fields, altered wall rock formed by the intrusion of magma and hydrothermal fluids, and very low-grade metamorphic rocks. Smectites exist as either dioctahedral or trioctahedral clay minerals, depending on whether two out of three cation positions in the octahedral sheet are occupied by trivalent cations (e.g., Fe^3+^ or Al^3+^) or fully occupied by divalent cations (e.g., Mg^2+^ or Fe^2+^), respectively. The dioctahedral smectites can be split further into two major groups: (a) aluminum smectites (e.g., beidellite and montmorillonite) and (b) iron-rich smectites, including ferruginous smectites and nontronites [109]. The trioctahedral smectites contain minerals such as saponite and hectorite.

Nontronites, together with a large variety of intermediate Fe/Mg smectites, can be found in oceanic as well as continental settings associated with basaltic rocks. Smectites found in subsurface settings have considerable amounts of ferrous [Fe(II)] iron instead of the usual ferric [Fe(III)] smectites that are found in soils, in terrestrial sediments, and on the ocean floor, where there is enough dissolved O_2_. The crusts found on planets in our solar system, such as Mars, are usually dominated by rocks of mafic and ultramafic compositions (low in Si, high in Fe and Mg), while the majority of the nonterrestrial bodies lack an oxidizing atmosphere. Actually, it has been suggested by some scientists that the early Martian atmosphere was anoxic, similar to the early anoxic atmosphere on Earth, for which there is extensive evidence [110,111,112]. Therefore, Fe/Mg smectites with intermediate compositions, with Fe(II), Fe(III), or combinations of both, can be anticipated to be common clay minerals formed on these types of bodies [113,114].

Smectite-containing deposits have been found on the surface of Mars through the use of orbital infrared instruments [24,27,30,36,37,115,116] and, in addition, have been studied on the surface by the MER Opportunity rover [117,118] and the Mars Science Laboratory (MSL) Curiosity rover (see Section 4) [34,35,119,120,121]. Fe- and Mg-containing smectites are the most frequently found clay minerals on the surface of Mars and have been detected in the majority of outcrops exposing ancient crust, pointing to early geological conditions favorable for silicate weathering or hydrothermal alteration [24,30,36,37,38,111,116,122,123]. In addition, Fe- and Mg-containing clay minerals, including smectites, have been observed in carbonaceous chondrite meteorites as well as on other altered bodies in our solar system, e.g., Ceres, C-class asteroids, and comets [58,59,124,125].

Martian clay minerals have been shown to resist laboratory-induced thermal shock, providing evidence that the clay minerals on the surface of Mars could have formed before meteorite impacts and survived, as water was more abundant more than 3.6 billion years ago [126]. Research on alteration products of nontronite and montmorillonite formed at temperatures between 350 and 1150 °C produced similar results [127,128]. The presence of Fe^3+^- and Mg^2+^-smectite, particularly in lacustrine deposits, is an indication that habitats that may have supported life are present on Mars [129].

### 5.1. Nontronite and Related Iron-Rich Smectite Synthesis

Smectites form one of the most significant mineral groups within the phyllosilicates (layer silicates) present in soils and sediments, and they are definitely some of the most complex to investigate. New data covering the development mechanisms, the influence of structural characteristics on the surface properties of smectites, and the long-term stability of smectites may be obtained through methodical research of phase-pure clay minerals. In nearly all cases, this type of sample can only be obtained via synthesis under controlled temperature, pressure, and other conditions. Over the past decades, the synthesis of various smectite minerals has been attempted (1) at ambient pressure and low temperature (<100 °C), (2) under moderate hydrothermal conditions (100–1000 °C, pressures of several kbars), (3) under extreme hydrothermal conditions (>1000 °C or pressures >10 kbars), (4) in the presence of fluoride, and (5) under microwave conditions [130].

Some of the earliest low-temperature syntheses of iron-containing clay minerals were performed by Caillère et al. [131,132], who formed nontronite and iron-rich saponite through the aging of mixed dilute solutions of silica, Fe^2+^ or Fe^3+^ chlorides, and Mg and Al salts at a temperature of 100 °C and a pH between 8.5 and 9.5. In the clay minerals obtained, the octahedral sheet was partially filled with either Mg or Fe^2+^. Harder [133,134], Decarreau and Bonnin [135], and Decarreau et al. [136] presented many low-temperature syntheses of Fe-containing smectites. The synthesis of nontronite under reducing conditions used by Harder [133] was similar to the method employed to synthesize different trioctahedral smectites [137,138]. The use of Na dithionite or hydrazinium dichloride created reducing conditions in the system. Decarreau and Bonnin [135] and Decarreau et al. [136] formed ferric smectites by applying a method comparable to that used to synthesize hectorite and stevensite [139]. This method encompassed the aging of freshly formed coprecipitated gels of silica with FeSO_4_ under initially reducing conditions at a temperature of 75 °C for a period of 15 days or 1 month, at a temperature of 100 °C for a period of 1 month, or at a temperature of 150 °C for a period of 12 days. After oxidizing the Fe^2+^ in the system, smectite crystallization was enhanced. Under only oxidizing conditions, Decarreau et al. [136] succeeded in synthesizing ferric smectites at 100 °C and 150 °C. This particular smectite was believed to represent a “defect” nontronite in which octahedral vacancies were responsible for creating the layer charge.

The earliest high-temperature nontronite synthesis attempts were published by Ewell and Insley [140] by reacting mixtures of silica gel with Fe_2_O_3_ at a temperature between 340 and 350 °C and a pressure of 167 bars for a period of 6 days. A mixture of nontronite, hematite, and another unidentified phase was formed. Hamilton and Furtwängler [141] reacted dilute solutions of NaSiO_3_ with FeCl_3_ at high temperatures to form nontronite. Mizutani et al. [142] produced Fe-rich 1:1 and 2:1 clay minerals through a hydrothermal reaction of a mixture containing silicic acid, FeSO_4_, and NaOH at temperatures between 100 and 200 °C for 50 h. The initial Fe/Si ratio defined whether 1:1 or 2:1 clay minerals were produced. Ratios of 0.75 and 1.5 produced nontronite-like materials. Nagase et al. [143] succeeded in synthesizing Fe-smectites at 100 and 200 °C for 24 h, starting with hydrous oxides formed by mixing acidic sodium silicate, FeCl_3_, and MgCl_2_ solutions, precipitated at pH = 9.9. Smectites were found in a narrow pH range, only from 12.0 to 12.4. When the pH was below 12, only an amorphous phase was observed, and when the pH was above 13, a combination of aegirine and hematite was formed.

A considerable amount of research has focused on the structural behavior of smectite-group clay minerals, as the composition changes along binary axes when synthesizing smectites with carefully controlled overall compositions as well as octahedral cation ratios. The Fe(III)-Mg (nontronite–saponite) series form a complete solid solution series when Al is absent [144], as does the Fe(III)-Al (nontronite–beidellite) solid solution series [145]. Grauby et al. [144] studied heterovalent octahedral Fe3+/Mg substitution via the hydrothermal synthesis of a smectite series at 200°C from starting gels having a theoretical stoichiometry of ^4^(Si_4_)^6^(Fe^3+^_(2–2x/3)_Mg_x_)O_11_, with x varying from 0 to 3. Synthesis research can limit the physical parameters, e.g., temperature, pressure, and pH, that govern the cation solubility within the layer structure of the smectite for binary solutions [146,147]. Decarreau et al. [146] hydrothermally produced high-charge ferric nontronite at temperatures between 75 and 150 °C for 4 weeks and, for the first time, precisely characterized the ferric end-member of nontronite. The hydrothermal synthesis started from a silico-ferrous starting gel, Si_2_FeNa_2_O_6_.nH_2_O, formed by combining sodium metasilicate and ferrous chloride solutions. The pH was adjusted to 12.5 by NaOH. Irrespective of the synthesis temperature, all nontronites formed had an identical structural formula of Na^+^_0_._75_^4^(Si_3_._25_Fe^3+^_0_._75_)^6^Fe^3+^_2_O_10_(OH)_2_.

Andrieux and Petit [147] were the first to hydrothermally crystallize Al-Fe^3+^ smectites within a broad compositional range with the general structural formula of Na_x_^4^(Si_4–x_(Fe^3+^-Al)_x_)^6^(Fe^3+^_2–y_Al_y_)O_10_(OH)_2_. The ranges of experimental parameters to form these smectites turned out to be rather narrow. Temperatures at or below 150 °C and high pH (∼12) can be used for the synthesis of Fe^3+^-rich smectites (nontronite), whereas higher temperatures (200 °C) and lower pH (down to 7) are better suited to synthesize most Al-rich smectites (Fe^3+^-beidellites). In order to obtain intermediate compositions, both the synthesis pH and temperature must be adjusted in parallel to favor the formation of pure smectite.

Baldermann et al. [148] hydrothermally synthesized Mg-Fe^2+^ saponites under reducing conditions (in the presence of 0.05 mass% of Na dithionite) at 120 and 180 °C for 2, 5, or 7 days, and the final pH was measured to be around 12.6. Initial precipitates were formed from mixing solutions of Na orthosilicate (Na_4_SiO_4_), FeSO_4_·6H_2_O, and MgCl_2_·6H_2_O to achieve initial molar Si:Fe:Mg ratios of 4:0:2, 4:1:1, 4:1.5:0.5, 4:1.75:0.25, and 4:1.82:0.18. The starting pH was adjusted to 8.5 using NaOH. The tetrahedral charge of thus-formed saponites, due to Si^4+^ by Fe^3+^ substitutions, was between 0.03 and 0.26 electrons (based on O_10_(OH)_2_), while the octahedral sheet consistently included Mg, Fe^2+^, and Fe^3+^ cations with a ^6^(Fe^2+^/Fe^3+^) ratio of around 10. Baldermann et al. [148] indicated that a complete octahedral substitution between Mg and (Fe^2+^ + Fe^3+^) cations may take place in ferrous saponites.

Examining the improvement in the hydrothermal synthesis method of Decarreau et al. [146], Baron [149] managed to successfully form nontronites from starting gels prepared from FeCl_2_, FeSO_4_, or FeCl_3_ salts. The particle size of the nontronite formed was more homogeneous when hydrated gels were employed as the starting materials instead of dehydrated powders. Baron [149] also hydrothermally synthesized nontronite in a microwave oven. At a temperature of 150 °C for 6 days, no variation in the amount or the crystal chemistry of the nontronites formed was observed between the microwave and classical hydrothermal methods. Baron et al. [150] managed to hydrothermally form a series of strictly ferric nontronites with different tetrahedral charges using starting aqueous pH values ranging from 11 to 14 at 150°C. The permanent negative layer charge of these nontronites, caused by the Fe^3+^-for-Si^4+^ tetrahedral substitutions only, ranged between 0.43 and 1.54 based on the structural formula unit of O_10_(OH)_2_. Notwithstanding the uncommon values of the layer charge, synthetic Ca-saturated nontronite layers have been shown to expand after solvation with ethylene glycol. The increase in tetrahedral Fe^3+^ has been linked to the increase in the final pH, measured at 25°C, after the synthesis was finished [151]. A similar trend was found when the data were recalculated for 150 °C. High pH values caused a sharp increase in SiO_2_ in solution through the formation of anionic aqueous Si species (e.g., H_3_SiO_4_^−^(aq) and H_2_SiO_4_^2−^(aq)), increasing the substitution of Fe^3+^ for Si^4+^ on the tetrahedral sites of these synthetic nontronites.

Fox et al. [151] synthesized a series of smectites that covered the intermediate compositional region between Fe(II), Fe(III), Mg, and Al end-member clay minerals by employing a hydrothermal sol–gel process adapted from earlier papers [135,152]. After pH correction to the required value, the freshly prepared gel suspensions were put in PTFE-lined Parr acid digestion vessels and hydrothermally treated in a furnace at a temperature of 200 °C for a period of 15 days. To avoid oxidation during the synthesis, the syntheses with Fe(II) were performed hydrothermally in a vacuum furnace containing ultrahigh-purity nitrogen gas.

### 5.2. Effect of Small Organic Molecules on the Synthesis of Smectites

Several recent studies have highlighted that not only do smectite-group clay minerals play a role as catalysts in organic reactions, but small organic molecules can also play an active role in the crystallization of clay minerals. The catalytic power of clay minerals, especially in the smectite group, can stimulate the polymerization and other organic reactions of biomolecules and the transformation of fatty acid micelles into vesicles [153]. Syntheses at low temperature at 1 atmosphere have shown that organic molecules, e.g., oxalate and urea, are able to catalyze the formation of clay minerals belonging to the smectite group [154,155]. Schumann et al. [154] succeeded in the formation of saponite, an ^IV^Al- and ^VI^Mg-rich trioctahedral smectite, starting with a silicate gel at a temperature of 60 °C and ambient pressure, in which oxalate acted as a catalyst. The fact that saponites with variable negative layer charge crystallized from the same starting gel has consequences for how we might be able to understand the origin of life, because smectites possibly replicate following a process of template-catalyzed polymerization while transferring the charge distribution from one layer to the next. Assuming that polar organic molecules such as oxalate are able to catalyze the crystallization of clay minerals such as saponite, they can then promote clay microenvironments and the creation of many different adsorption sites for other organic molecules present in solution within the smectite’s interlayer spaces and on external surfaces as well as on the edges. The reaction between adsorbed molecules could result in the formation of more complex organic compounds, such as RNA from nucleotides, on early Earth, Mars, or other bodies containing organic molecules and clay minerals in our solar system.

Zhou et al. [156] reported the crystallization of clay minerals catalyzed by succinate (and other organic acids), a good example of a photoproduced intermediate from central metabolism. This study connected the formation of sauconite, which can act as a model for other clay minerals such as saponite, to prebiotic photochemistry. It also proved that seeding with only a single clay particle induced nucleation at low temperatures, thereby increasing the speed at which crystallization took place. The co-catalytic function of succinate in the interlayer spaces of sauconite was thought to produce soluble Al^3+^ complexes, which form the limiting reagent in sauconite crystallization. After the intercalation of succinate (or another organic species) has balanced the relative adsorption forces between the precursor gel and the Al species, because of its bidentate ability, it can reverse the attractive forces among neighboring sauconite whiskers. The effect of the nature of the organic salt was studied through the substitution of succinate with sodium salts of formic acid, acetic acid, oxalic acid, and malic acid. In all cases, except for oxalic acid, a low degree of stacking of the sauconite layers was observed. The addition of a single crystal of sauconite acting as a seed to crystallize a larger quantity of sauconite provides an excellent illustration of the self-catalytic power of clay minerals with respect to their formation. The seed crystal’s surface accelerated heterogeneous nucleation at a lower temperature, as it decreased the activation energy for crystallization. The detected increase in crystallization through seeding is not only dependent on random events but also caused by the surface interaction of the clay particle with chemical species present in the starting gel. The seed particle interacts with soluble free and complexed ions moving freely through the gel, creating intermolecular forces that are required to create the clay layer structure. Unfortunately, this is an area of research that has not yet received the attention it deserves. It could potentially explain a lot about the formation of clay minerals in nature.

Recently, a review by Ponce and Kloprogge [155] reported the use of urea in the synthesis of saponite based on the work by Vogels et al. [157]. Urea is a compound that is readily formed through cyanide hydrolysis and is frequently employed as a model molecule in traditional prebiotic reactions. The structure of urea and its properties make it a theoretically good initial compound to form nucleobases and associated compounds [158,159]. In addition, it has been proven to help phosphorylation reactions [158,159]. Furthermore, it has been shown that it can catalyze the crystallization of saponite in just 20 h under relatively mild temperature conditions [157]. The crystallization process developed by Vogels et al. [157] broke the synthesis down into two steps: (a) the production of the starting aluminosilicate gel and (b) the hydrolysis of the divalent metal controlled through the gradual release of ammonia during the decomposition of urea, serving as a fair equivalent to naturally occurring processes. The initial aluminosilicate gels contain tetrahedrally coordinated Al^3+^ comparable to fourfold coordinated Al^3+^ present in natural systems, such as in volcanic glass. The utilization of urea prevents the crystallization of brucite, Mg(OH)_2_, during the synthesis through the slow release of ammonia during the urea breakdown, thus limiting the hydrolysis of Mg^2+^ [160]. This method allows the crystallization of saponite without the associated abrupt increase in pH resulting in the precipitation of brucite [160]. Besselink et al. [161] showed that for the crystallization of saponite, it was necessary to form an intermediate phase first, which they demonstrated using a pair distribution function analysis on samples obtained from syntheses quenched at different times during crystallization. The existence of an intermediate phase was further proven using transmission electron microscopy, which showed the existence of amorphous spherical globules prior to their subsequent conversion in the synthesis into smectite-like layers with well-defined basal spacings.

### 5.3. Smectites: Catalytic Organic Reactions and Other Organic Interactions

**Scheme 1:**
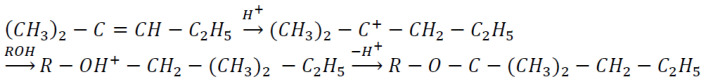
An example of an acid-catalyzed reaction of 2-methyl pent-2-ene with alcohol to 2-methyl-2-alkoxy pentane over Al-montmorillonite catalyst.

Clay minerals have a high tendency to take up organic molecules and act as a catalyst for numerous organic reactions because of their small particle size, large surface area, layer structure, and unusual charge properties. Clay minerals can act as heterogeneous catalysts for organic reactions in various ways [162]. Clay minerals can stabilize high-energy intermediates and can store energy in their layer lattice structures, subsequently releasing it as chemical energy. Clays can act as redox catalysts and can also serve as photocatalysts. Clay minerals frequently have a high surface acidity [163]. The presence of high acidity has been described as being due to the occurrence of exchangeable cations in the interlayer space. These interlayer exchangeable cations will polarize the coordinated water molecules in the interlayer space and cause their dissociation. This process clarifies the reduction in surface acidity with increasing humidity. Alkenes can easily be protonated in the interlayer space of smectites. Because the interlayer Brønsted acidity of a smectite decreases with the charge of the exchangeable cation, it is highest with trivalent ions, e.g., Cr^3+^, Al^3+^, and Fe^3+^ [164,165]. The carbocations formed can further react with interlayer water to produce alcohols and ethers [162,166,167]. In addition, alcohols can react with alkenes (Scheme 1), particularly if they replace water in the interlayer space, to form ethers [168,169,170].

**Scheme 2:**
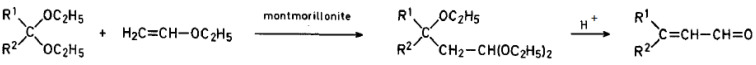
Clay-catalyzed reaction of acetals with enol ethers to produce precursors of α,β-unsaturated aldehydes. R^1^ = C_6_H_5_, CH_3_, C_2_H_5_, n-C_6_H_13_, or -(CH_2_)_5_-; R^2^ = H or -(CH_2_)_5_-.

Various additional carbocationic reactions are known to be catalyzed by acidic clay minerals [171]. Acetals can react with enol ethers to produce precursors of α,β-unsaturated aldehydes [172,173,174] (Scheme 2). Esters are easily formed via the direct addition of carboxylic acids to alkenes [167,175].

**Scheme 3:**
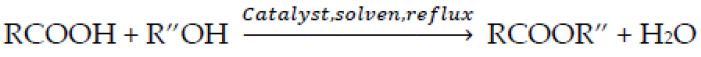
Esterification of alcohol with montmorillonite catalyst. R = alkyl or aryl or substituted aryl; R” = primary or secondary alcohol.

**Scheme 4:**
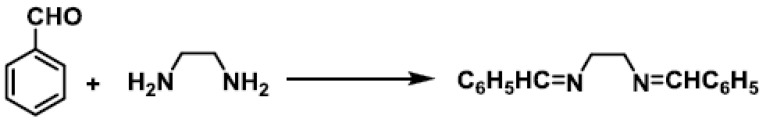
Synthesis of N^1^,N^2^-dibenzylideneethane-1,2-diamine using montmorillonite as catalyst.

Clay minerals can be employed as solid acids for several reactions typically catalyzed by mineral acids in aqueous solutions, e.g., the esterification of carboxylic acids [176,177,178,179,180,181] (Scheme 3), lactone production [182,183,184], and the formation of amines, amides, enamines, and amino acids [185,186,187,188,189,190] (Scheme 4).

**Scheme 5:**
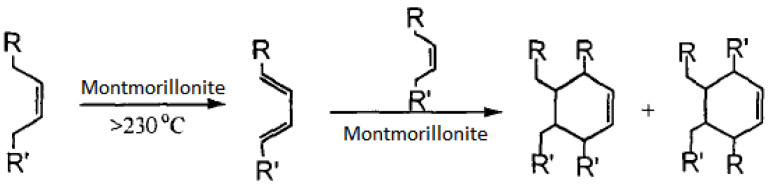
An early Diels–Alder reaction is the dimerization of oleic acid on montmorillonite. (It is presumed that only the first step of dehydrogenation is catalyzed by montmorillonite, but not the Diels–Alder addition part itself.)

Adsorption on the different surfaces of a clay mineral lowers the dimensionality of the reaction space from 3 to 2. This restriction results in greatly enhanced reaction rates. Several Diels–Alder cycloadditions are catalyzed by Lewis acids and are sensitive to clay mineral catalysts [167,173,191,192] (Scheme 5).

**Scheme 6:**
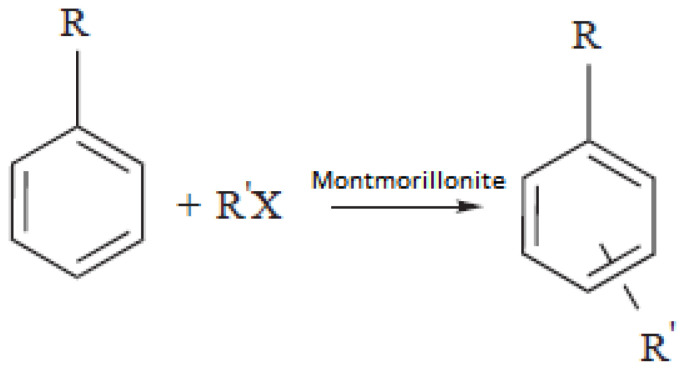
Montmorillonite-supported transition-metal salts (zinc and nickel chlorides) catalysis of Friedel–Crafts alkylation.

Another important organic reaction is formed by the Lewis acid–catalyzed Friedel–Crafts reaction [162,167,173,174,193,194,195] (Scheme 6).

Reactions catalyzed by Lewis acids will likewise be catalyzed by Lewis acidic clay minerals, with the increased reaction rate resulting from the reduction in dimensionality from 3 to 2. A clay surface with the associated counterions in the electrical double layer can be envisaged as the equivalent of a cathode immersed in an electrolytic solution but without the continuous electric current. Consequently, redox reactions can easily take place on clay mineral surfaces as long as energy is available. The necessary energy can be generated by the reorganization of the clay layer structure with a general reduction in the potential energy. Finally, facile oxidation of aromatic compounds on clay mineral surfaces into their analogous radical cations has made different reaction paths for their nitration possible [162,173,196,197,198].

**Scheme 7:**
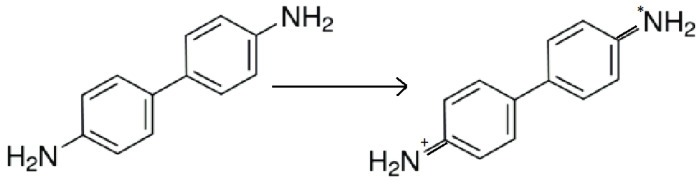
Oxidation of benzidine to benzidine blue catalyzed by nontronite. Benzidine blue is a radical cation, and the blue color indicates that both aromatic rings are involved in the resonance structure.

The amount of research on the catalytic properties of nontronites in particular is much more restricted. The oxidation state of structural Fe in smectites can significantly change its surface chemistry and may result in a considerable effect on smectite–organic interactions. Structural Fe(III) found in nontronite is generally considered a Lewis acid (an electron acceptor) in oxidation–reduction reactions [199]. Nevertheless, the power of nontronite as a catalyst in one of the first studies was solely determined visually based on its ability to oxidize benzidine to benzidine blue [199] (Scheme 7).

**Scheme 8:**
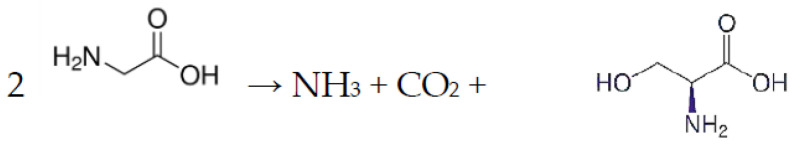
Decarboxylation and deamination of glycine to serine.

Ca-exchanged nontronite can act as an abiotic catalyst in the oxidative polymerization of hydroquinone, resulting in the transformation into various phenol-derived macromolecules, some of which bear a resemblance to naturally occurring humic compounds. The polymerization of hydroquinone in the presence of Ca-nontronite was observed to be substantially higher in air in comparison to a N_2_ atmosphere, suggesting a synergetic effect between Ca-nontronite and O_2_ in improving the reaction. Both Fe(III) on the edges and other Lewis acid sites of Ca-nontronite were thought to support the polymerization of hydroquinone [200]. In a follow-up study, the same scientists reported on the use of Ca-nontronite and Ca-bentonite together with kaolinite as heterogeneous catalysts in the abiotic ring cleavage of pyrogallol (1,2,3-trihydroxybenzene) with the accompanying production of humic polymeric compounds in the absence of microbial activity [201]. Using a starting pH of 6.00 and a reaction time of 90 h, the volumes of CO_2_ produced during the ring cleavage of pyrogallol as well as the amounts of the humic polymeric compounds produced in the catalytic reactions were in the order of: Ca-nontronite > kaolinite > Ca-bentonite. This suggests that the catalytic activity of Fe(III) on the edges and within the layer structure of nontronite was significantly higher compared to that of Al present at the edges of kaolinite and montmorillonite and of a minor amount of Fe(III) present in the crystal lattice of montmorillonite in supporting the catalytic reaction. Wang [202], in a comparable study, reported on the use of Ca-nontronite as a solid catalyst for the polycondensation of phenols and glycine and the related reactions involving the ring cleavage of phenols and the decarboxylation and deamination of glycine in the absence of microbial activity. The formation of CO_2_ and NH_3_ in the Ca-nontronite–glycine interaction showed that Ca-nontronite acted as a catalyst for the decarboxylation and deamination of glycine (Scheme 8).

The capacity of Ca-nontronite as a heterogeneous catalyst for the deamination of glycine was significantly increased when phenol was present.

Cervini-Silva et al. [203] studied the influence of the structural Fe oxidation state on chlorinated hydrocarbons present at the smectite–water interface. Pentachloroethane was reacted with a small set of smectites, comprising montmorillonite, ferruginous smectite, and nontronite, suspended in water using restricted atmospheric conditions. Pentachloroethane adsorbed on the three different smectites at various rates. A set of pentachloroethane adsorption rate constants on the three smectites exhibited a clear relationship with respect to the amount of Fe(II) present in the smectite layer structures. The smectite surface acts as a Brønsted base and promotes pentachloroethane dehydrochlorination. The Brønsted basicity in smectites can be enhanced through structural Fe reduction from Fe(III) to Fe(II). Furthermore, the Fe reduction increases the surface charge density of the smectite [204,205], causing the Fe(II)-bearing smectites to develop a strong nucleophilic nature that assists in the catalytic reaction of chlorinated alkanes.

**Scheme 9:**
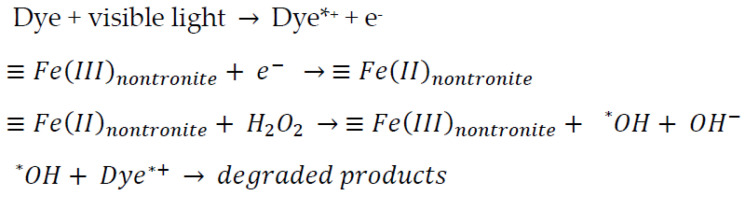
Essential reactions in the catalytic breakdown of a dye such as Rhodamine B with H_2_O_2_ and nontronite as solid catalyst.

The traditional photo-Fenton reaction is frequently adversely affected by the constraints of operating conditions such as pH, low Fe concentration, the amount of ultraviolet light available in sunlight, and the instability of Fe-based catalysts. Liu et al. [206] published a new heterogeneous Fenton reaction using a dye-photosensitized structural Fe(III)/Fe(II) redox cycling method. Synthetic nontronite showed good catalytic activity over a pH range between 3.0 and –8.0 for the extremely effective breakdown of Rhodamine B by H_2_O_2_ using irradiation with visible light. The excited dye molecule acted as a donor of electrons to structural Fe present in the nontronite, which then catalyzed H_2_O_2_ to form very reactive *OH radicals (Scheme 9).

The nontronite was shown to be both chemically and mechanically stable under the reaction conditions. After six cycles, no measurable Fe leaching or damage to the nontronite structure was detected, nor was any detectable activity loss observed. The Fenton reaction serves as an effective process for the removal of refractory organics, e.g., carbamazepine, from wastewater streams. However, its use is significantly compromised due to the addition of extra H_2_O_2_ and the accumulation of iron mud. Shi et al. [207] used a Fenton-like process with in-situ formed H_2_O_2_ by biosynthesized palladium nanoparticles in combination with a natural nontronite for the degradation of carbamazepine. Oxidative radicals such as HO* together with H_2_O_2_ played crucial roles in the degradation of carbamazepine. The identification of intermediates/products and theoretical calculations provided evidence that hydroxylation was mainly responsible for the major carbamazepine breakdown.

Zhang et al. [208] hydrothermally synthesized nontronite and used it as a heterogeneous catalyst for the activation of bisulfite for the degradation of tetracycline. Due to structural Fe(III) in the nontronite catalyst, it exhibited good catalytic activity and low Fe leaching in the pH range between 3.0 and 7.5. The nontronite particles were shown to be stable and could be reused in the activation of bisulfite for the breakdown of tetracycline. Based on EPR and radical quenching tests, they showed that the precursor radical *SO3^−^ was initially formed in the nontronite/bisulfite system, followed by *SO4^−^ and *OH as the active species taking part in the tetracycline breakdown.

Recently, several papers have been published that focus on the role of nontronite and its interactions with organics related to the origin of life and under conditions found on Mars. A number of publications have previously concentrated on the formation of pure organic molecules under experimental surface conditions on Mars, e.g., [209,210,211,212,213,214,215,216], but only a limited number have assessed the impact of the mineral matrix [217,218,219]. Poch et al. [220] qualitatively and quantitatively described the reactions of glycine, urea, and adenine in contact with nontronite, one of the most common smectite minerals found on the surface of Mars, using virtual Martian surface ultraviolet light (between 190 and 400 nm), average temperature (−55 °C ± 2 °C), and average pressure (6 ± 1 mbar) conditions. They examined organic-rich samples that were thought to correspond to those present after the evaporation of a small, warm pool containing liquid water with an elevated concentration of organic compounds. They found a distinct photoprotective influence of nontronite on the release and breakdown of glycine and adenine, with the efficiencies of photodecomposition decreased five-fold after mixing at a concentration of 2.6 × 10^−2^ mole/g of nontronite. Additionally, after the volume of nontronite in the glycine sample was doubled, the photoprotection again increased five-fold. These results suggest that photoprotection by nontronite is not only due to mechanical shielding but also caused by stabilizing interactions. Other reaction products were not detected with certainty, though the results found for urea may indicate a certain reactivity with nontronite, resulting in an increase in its dissociation rate. Gil-Lozano et al. [221], in a comparable study, assessed the capability of nontronite to protect organic compounds affected by a brief period of contact with various diagenetic fluids. They examined the stability of glycine mixed with nontronite previously treated with either acidic or alkaline fluids under conditions thought to be present on the surface of Mars. Their results indicated increased photodegradation of glycine on the acid-treated nontronite, caused by decarboxylation and deamination reactions. On the other hand, the experiments with alkali-treated nontronite revealed that glycine was preferably adsorbed through ion exchange in the nontronite interlayer spaces, thereby better protecting the glycine from external conditions. This shows that previously subjecting nontronite to fluids with different pH values determines the manner in which glycine is adsorbed into the nontronite’s interlayer spaces, influencing the possibility for the conservation of organic molecules under the current and rather harsh surface conditions on Mars.

## 6. Smectite and the Origin of Life

From the previous sections, it is clear that clay minerals, particularly the smectite group, including nontronite, are present on a variety of different bodies in our solar system and can function as catalysts for a large variety of organic reactions. In addition, a large number of organic compounds have been found on these bodies as well. Simple to rather complex organic molecules have been observed on Mars [222,223,224,225,226,227,228,229,230,231], on the Moon [232,233], in comets [234,235,236,237,238], in meteorites [239,240,241,242,243,244,245,246,247,248,249,250,251,252,253,254,255,256,257,258,259,260,261,262,263,264,265,266,267,268,269,270], and in asteroids [237,267,271,272,273,274,275]. Though much smaller volumes of organic compounds have been found on Mars than expected, this does not mean that they were not present at an earlier stage in the geological history of Mars. The fact that significant amounts and a variety of organic compounds have been found on Martian meteorites show clear evidence for this [268,276,277,278,279,280]. It is highly likely that the presence of strongly oxidizing compounds, such as perchlorate, and the intense UV and high-energy particle radiation [281] may have been responsible for the lack of a large volume of organic compounds currently detected on Mars.

The presence of organic molecules, water, and smectite minerals potentially creates ideal conditions in which the clay mineral surface can act as a catalyst for the formation of more complex organic molecules and as a protector of the organic molecules from outside influences, such as exposure to intense radiation and oxidizing molecules. Existing data on heterogeneous catalysis by smectite clay minerals typically come from industrial processes that have to be extremely fast (around seconds to minutes) to be efficient. Consequently, these reactions have to be performed at relatively high temperatures, doubtful to have been reached in most circumstances on early Earth (see Section 5.3). Nevertheless, it is not hard to extrapolate reaction rates to lower temperatures, and numerous catalytic reactions will still be fast enough under more moderate temperatures and pressures when taking into account a geological time scale [282].

Bernal [283] first pointed to the potential importance of clay minerals in the origin of life due to the well-ordered arrangement of clay mineral particles, their high adsorption capacity, their ability to shield molecules from ultraviolet radiation, their capacity to adsorb and concentrate organic molecules, and their ability to act as polymerization templates. The idea that clay minerals acted as heterogeneous catalysts for the precursor organic molecules for the origin of life was first seriously expanded upon by Cairns-Smith [9] and has found significant traction since. According to him [284], there is no persuasive reason that a relationship is required between the last common precursor consisting of organic molecules and initial life on Earth. While the easy availability of a large variety of organic building blocks of life has been shown in laboratory experiments, the major use of these organic compounds in living systems can be interpreted as being caused by evolution instead of a requirement for its onset. He suggested that the initial living organisms and the chemical evolution before them might have been centered on a chemistry that was completely distinct from what is seen now. The complex genetic systems currently found in living organisms evolved secondarily in a living organism employing a less effective primary system with a far better chance of spontaneous assembly. As genetic possibilities, Cairns-Smith proposed naturally occurring crystalline inorganic materials, i.e., minerals, with appropriate properties, e.g., the capacity to store and replicate information through defaults, dislocations, and substitutions. Clay minerals, e.g., kaolinite- and smectite-group minerals, are especially interesting since these clay minerals can form at relatively low temperatures in aqueous solutions produced from weathering products from various silicate rock types.

Bernal’s idea about the function of clay minerals in the initial phases of chemical evolution on the surface of early Earth can be expanded with the following steps:

1. Clay minerals acted as solid catalysts for reactions in biomonomer synthesis from the gaseous molecules present in the primordial atmosphere and in water on the Earth’s surface.

2. Clay minerals acted as high-surface-area and porous materials and adsorbed these newly formed biomonomers on their surfaces and in their interlayer spaces, resulting in a highly concentrated system in which these biomonomers have to orient themselves in a particular direction.

3. Clay minerals acted as catalysts for condensation reactions between adsorbed biomonomers to form biopolymers. Moreover, the charged external and internal surfaces of clay minerals could have acted as templates for the specific adsorption and replication of organic compounds [285].

Weiss [286] initially seemed to offer experimental proof for Cairns-Smith’s idea. He used a “matrix” montmorillonite (Mt) with a negative layer charge of 0.28 charges/(Si,Al)_4_O_10_. An amount of Mt was suspended in a starting solution with Na^+^, K^+^, Mg^2+^, Al^3+^, and Si(OH)_4_. The cation concentrations of the starting solution were chosen so that homogeneous nucleation without matrix layers formed Mt with isomorphous substitution, equivalent to a negative layer charge of 0.42 charges/(Si,Al)_4_O_10_ unit. The first-generation Mt had a net negative layer charge of 0.28 charges/(Si,Al)_4_O_10_. This first-generation Mt was subsequently employed as a matrix for the corresponding formation of the second-generation Mt. Until the 10th-generation Mt, similar results were obtained, with the primary product being Mt having a negative layer charge of 0.28 charges/(Si,Al)_4_O_10_. After that, the number of errors increased rapidly with each further generation. In the 20th generation, nearly no Mt with the original value of 0.28 charges/(Si,Al)_4_O_10_ unit persisted. Despite the fact that a rapid decay in replication quality occurred in later generations, it showed that clay minerals were able to replicate. Unfortunately, various requests for specifics about the exact experimental setup were presented to the author in order to replicate his experiments, but no adequate response was ever received [287]. Therefore, the clay-mediated replication at that time could not be judged as proven.

### 6.1. Simple Biomolecules

**Scheme 10:**
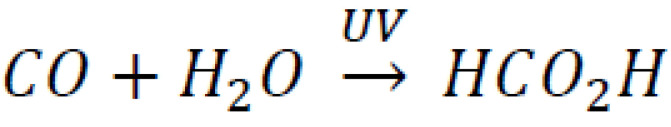
Formation of formic acid from CO and H_2_O.

Prebiotic syntheses using gas compositions varying between strongly reducing and mildly oxidizing in combination with several different energy sources in the presence of clay minerals have successfully formed organic monomers, e.g., amino acids and/or nucleic acid bases, crucial for living organisms. In some synthesis setups, a small volume of water was adsorbed on the surface of clay minerals, whereas in other experiments, the clay minerals were suspended in water, a situation thought to simulate the primordial ocean. The first condition, in which only a small amount of water is available, increases the surface acidity of the clay mineral particles as a result of the dissociation of the adsorbed water molecules. Increased acidity is important for catalytic reactions occurring at or near the clay mineral surfaces. Model syntheses utilizing clay minerals were described by Yoshino et al. [288], who detected eight different amino acids using Mt and a gas mixture of H_2_, CO, and NH_3_ (though a significant amount of adsorbed water must also have been present) after 5 h at 700 °C. Shimoyama et al. [289] executed experiments employing an electric discharge in a gas mixture of CH_4_ and N_2_ in the presence of Na-Mt suspended in water. The amino acids formed were mainly glycine, alanine, α-aminobutyric acid, and sarcosine, while serine, glutamic acid, valine, isoleucine, β-alanine, and α-, β-diaminobutyric acid were also detected. Alanine and α-aminobutyric acid were found to be racemic mixtures. Hubbard et al. [290] detected the production of formic acid and other organic molecules on Mt and other siliceous materials after mixtures containing CO, CO_2_, N_2_, and H_2_O were irradiated with UV light (250 nm) (Scheme 10).

Dry conditions with no residual water resulted in optimal adsorption of HCN on Ca- and Cu-exchanged Mt [291]. Heating the clay mixtures to 90 °C under dry conditions resulted in some differences in the infrared spectra, pointing to the creation of carbonyl or carboxyl bonds; however, the reaction products in this case were not well described. Ferris et al. [292] observed that Mt stopped the oligomerization of HCN. Additional experiments indicated that Mt catalyzed the decomposition of diaminomaleonitrile (DAMN) in aqueous solution, with the formation of two equivalents of HCN per DAMN, indicating that HCN oligomers and the derived biomolecules were probably not produced in significant amounts on the surface of early Earth with Mt present.

**Scheme 11:**
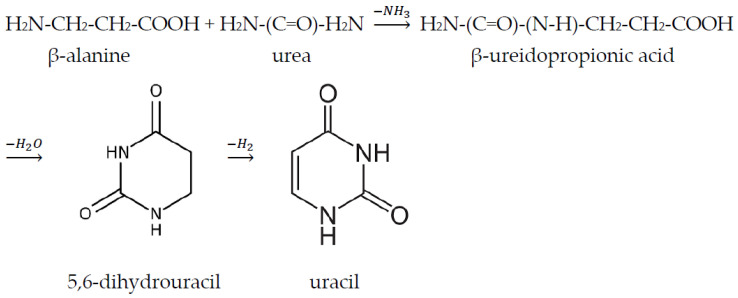
Reaction pathway for the formation of uracil from β-alanine and urea.

In order to form biologically important macromolecules, e.g., peptides and polynucleotides, from monomers, dehydration–condensation reactions are necessary, which would not be favorable under the conditions present during the early history of Earth. These biological macromolecules have lower thermodynamic stability compared to the monomers that they are formed from under aqueous conditions. Moreover, the presence of water will result in a shift in the reaction equilibrium in the direction of the monomers. The condensation reaction of amino acids may have been supported by various energy sources or chemical agents. While high energy input by ultraviolet light, electric discharge, and heat during the early history of Earth might have supported polymerization reactions, it would also have sped up the decomposition of the macromolecules formed. Nevertheless, adsorption of these macromolecules on clay mineral surfaces would have resulted in their protection from degradation to some degree. Fripiat and Cruz-Cumplido [164] reviewed some of the earliest experimental work in this area. Degens and Matheja [293] studied the catalyzed formation of peptides by using a large number of amino acids and a variety of solid catalysts, including clay minerals. They thought that the amino acid polymer was most likely formed via carboxyl activation. Adenine might be formed prebiotically from hydrogen cyanide, and cytosine might be derived from cyanoacetylene, whereas uracil might be formed from cyanoacetylene through malic acid (Scheme 11).

Chittenden and Schwartz [294] observed that the presence of Mt improved the adenine production rate. Nucleic acid bases were not detected in the initial experiment by Miller [2,295]. However, adenine was produced after a combination of HCN with Mt was added to the reaction flask and subjected to electric arcing [296]. Likewise, uracil was formed from CO, N_2_, and H_2_O using proton irradiation [297]. Replicating a simple fluctuating primitive geologic system, Lahav et al. [298] exposed combinations of glycine with kaolinite or Na-bentonite to several cycles of wetting and drying together with temperature fluctuations (25–94 °C). They detected the production of oligopeptides up to five glycine residues in length. Wetting and drying cycles, in addition to temperature fluctuations, increased the amount as well as the chain length of the peptide. It was proposed that the monomers and peptides had been rearranged on the clay mineral surface throughout the wetting and drying cycle, hence permitting better contact between monomers and peptides to allow for additional polymerization reactions. Others positively identified the creation of oligopeptides, up to decamers in some instances [299,300].

Williams et al. [301] found that some smectite clay minerals can transform simple carbon-based molecules such as methanol into complex ones in conditions simulating those found near high-temperature hydrothermal vents on the sea floor. Such complex organic molecules would have been crucial compounds in the first cell-like systems on Earth. They reacted montmorillonite (Mt), saponite, and illite with dilute methanol solutions as a source of C. Under these conditions, Mt was observed to transform into illite, whereas the saponite and illite did not show any alteration. Increased organic synthesis was detected with increasing experiment duration with Mt. Lower amounts of organic compounds were formed with saponite, and illite formed only very small volumes that were mainly found in the quenched gas phase. The most important molecule observed in the gas phase was dimethyl-ether with other major molecules, such as CH_4_, CO_2_, and H_2_. In addition, small amounts of alkanes and alkenes were detected. Mt and saponite formed a range of C7–C20 organic molecules, whereas illite only formed an extremely small amount of alkyl-benzene. Hexamethylbenzene was the most important organic molecule produced. When approaching equilibrium, the smectite contracted, and the newly formed organic molecules were expelled. It was suggested that organic molecules unstable in the hot fluid where the smectite reacted could possibly persist in lower-temperature waters outside of the hydrothermal vent.

### 6.2. Chirality

Amino acids occur as two enantiomeric (chiral) forms, i.e., D (dextrorotatory) and L (levorotatory). Both D- and L-enantiomers would have been produced in identical volumes under abiotic conditions, resulting in a racemic mixture with a D/L molar ratio of 1/1. However, the amino acids found in living systems are typically L-enantiomers. This selectivity in living systems forms one, if not the most important, of the major issues related to the origins of life [302]. Is it possible that clay minerals are able to differentiate between D- and L-amino acids when exposed to a racemic mixture? With Na-Mt in combination with a racemic mixture of various amino acids, Friebele et al. [303] found no preference in the adsorption of D- or L-enantiomers. This is not unexpected because clay minerals possess no chirality in their bulk layer structures, even though the lattice structure of kaolinite may possess some chirality because of the existence and location of vacancies. In addition, the edge surface of an Mt layer may show some structural chirality because of the occurrence of defects. However, these chiral structures are not individually separable. Siffert and Naidja [304] observed that Mt exhibited stereoselectivity in the adsorption and deamination of aspartic and glutamic acids. Similarly, Hashizume et al. [305] found that allophane exhibited a distinct preference for L-alanyl-L-alanine compared to its D-enantiomer. It was proposed that the size, intramolecular charge separation, and surface orientation of L-alanyl-L-alanine zwitterions combined to give “structural chirality” to the allophane–amino acid complex. While allophane was purified prior to its use, minor amounts of organic material could have resulted in the formation of the chiral “imprint” instead of allophane. Damien and Luthey-Schulten [306] used classical molecular dynamics calculations to obtain the atomic details of reactant conformations before they reacted to form polynucleotides. Their research assessing reaction probabilities concerning L- and D-chiral reactants showed the enhancement of homochiral products over heterochiral products when Mt was used as a catalyst.

### 6.3. Adsorption on Clay Minerals

Bernal [307] suggested that clay minerals could have concentrated organic molecules in the “primordial soup” via adsorption, as initially indicated by Haldane [5]. The adsorption of biologically essential organic compounds (e.g., amino acids, peptides, proteins, nucleic acid bases, nucleosides, nucleotides, polynucleotides, and sugars) by clay minerals has been investigated extensively over the past decades, e.g., amino acids on sediments and clay minerals [308,309], amino acids plus transition metals on clay minerals [310,311], adenosine triphosphate and related compounds plus metals on clay minerals [312,313], and nucleic acid bases on clay minerals [314,315].

McLaren et al. [316] observed that Mt could adsorb more alanine than expected based on its exchange capacity, whereas Sieskind [317] reported that the amount adsorbed could not reach the clay exchange capacity under comparable conditions. The difference in these results seems to be caused by the use of two different techniques to calculate the amount of adsorbed amino acid. McLaren et al. [316] found their results indirectly by determining the difference between the initial and the recovered amino acid concentrations in solution at equilibrium. In contrast, Sieskind [317] estimated the amount of adsorbed amino acid directly by determining the amount of amino acid retained in the clay residue after washing with water. In order to gain better insight into these differences, Cloos et al. [318] repeated both measurement methods, analyzing the amounts of amino acids in the equilibrium solution (indirect) and amino acids on washed clay residues (direct). The indirect method resulted in higher amounts of adsorbed amino acids. The disparity between these two techniques was partly explained by the weakly adsorbed amino acid molecules being desorbed in the washing step in the direct method.

The increase in the adsorption of amino acids with decreasing pH and increasing amino acid concentration suggests that the principal adsorption mechanism at low pH is cationic exchange, as the amino acids exist in their cationic form [317,318,319]. Friebele et al. [320] found another adsorption mechanism at low pH on Na^+^-Mt. The amount of amino acid that is strongly adsorbed (not removed by washing with water) on Mt at pH 3 increased with the increasing isoelectric point of the amino acid. Therefore, strongly adsorbed amino acids are adsorbed through cationic exchange. In contrast, the amount of weakly adsorbed amino acids (washed from Mt with pH-adjusted water) did not depend on the isoelectric point of the amino acid. H-bonding of the amino group to the oxygen atoms of water molecules in the interlayer spaces is a potential mechanism to explain adsorption for weakly adsorbed amino acids. Cloos et al. [318] provided two different mechanisms for the adsorption of amino acids on H^+^-Mt under neutral conditions: (1) proton transfer and (2) zwitterion association. The number of molecules that were adsorbed through the second mechanism and were desorbed from the Mt surface through washing with water followed the increasing K_1_ values of the amino acids. In primitive ocean environments, which might have been slightly alkaline because of dissolved ammonium ions [321], a substantial concentration of amino acids could have occurred by adsorption on clay minerals, even though the majority of the amino acids were present as zwitterions and only limited cation exchange took place. Greenland et al. [322] reported that cations were not liberated when amino acids and peptides were adsorbed on Na^+^- and Ca^2+^-Mt at pH 7. Greenland et al. [322,323] researched the interactions of several amino acids with H^+^-, Na^+^-, and Ca^2+^-Mt. Arginine, histidine, and lysine were adsorbed by Na^+^- and Ca^2+^-Mt through cation exchange. Different amino acids (alanine, serine, leucine, aspartic acid, glutamic acid, and phenylalanine) were adsorbed on H^+^-Mt through proton transfer. Adsorption of glycine, together with its oligopeptides, by Ca^2+^-Mt and Ca^2+^-illite was shown to increase with the degree of oligomerization.

Lawless et al. [324] and Banin et al. [325] observed that the amount of adsorbed adenosine monophosphate (AMP) by Mt, with various exchangeable interlayer cations (Zn, Cu, Mn, Fe, Ca, Co, and Ni), usually increased as the pH of the solution decreased. For Zn-Mt, the adsorption of 5′-AMP was optimal at around pH 7. The amount of adsorbed AMP was mainly affected by the acid dissociation constant of the nucleic acid base. Winter and Zubay [326] studied the relative absorption capacity of Mt and hydroxylapatite (Ca_5_(PO_4_)_3_OH) for adenine and adenine-related molecules. They determined that Mt was able to adsorb more adenine compared to the other molecules (adenosine, 5′-AMP, 5′-ADP, and 5′-ATP), whereas hydroxylapatite showed a preference for adenosine phosphate compared to adenine and adenosine. The amount of adsorption was dependent on the pH of the solution, though it might also have been influenced by the buffer utilized.

Hashizume et al. [327] studied the adsorption of adenine, cytosine, uracil, ribose, and phosphate on Mg-Mt. They found that at similar concentrations in the equilibrium solution, the adsorption on Mt decreased in the order adenine > cytosine > uracil, while ribose was barely adsorbed at all. Hashizume and Theng [328] observed that allophane possessed a higher affinity for 5′-AMP compared to adenine, adenosine, and ribose. Similar to Mg-Mt, barely any ribose was adsorbed by allophane. The high amount of adsorption of 5′-AMP agrees with allophane’s significant phosphate retention capacity [329]. Kalra et al. [330] studied the adsorption of alanine and glycine on Mt and its Mg^2+^ and Ca^2+^-exchanged equivalents at pH values between 4 and 9. Significant adsorption of both amino acids was detected for all three Mt samples. The highest adsorption was found at 25 °C and pH 7. Comparatively better adsorption of the amino acids was observed for Ca^2+^-Mt compared to Mg^2+^-Mt and the initial starting Mt. On all three Mt samples, the adsorption followed Langmuir-type adsorption, pointing to the formation of a monolayer of the amino acids on the Mt surface. In an investigation by Carneiro et al. [331], the adsorption of nucleic acid bases with sulfide-modified Mt was characterized at pH 2 and 7. The nucleic acid bases penetrated the interlayer spaces of Mt and oxidized Fe^2+^ to Fe^3+^. At the two pH values used in this study, the adsorption order of the nucleic acid bases on Mt was reported to be adenine ≈ cytosine > thymine > uracil. Adenine and cytosine adsorption on Mt increased when the pH was lowered. For non-exchanged Mt, the results may be explained by the electrostatic forces acting between the positively charged adenine/cytosine and the negatively charged layers of Mt. Infrared spectroscopy indicated that the interaction between the nucleic acid bases and Mt took place via NH^+^ or NH_2_^+^ groups. The nucleic acid bases adsorbed on Mt were detected in the interlayer spaces and on the external surface aluminol and silanol groups [331]. The adsorption of adenine on Mt with or without divalent cations Ca^2+^, Cu^2+^, and Mg^2+^ was studied by Gururani et al. [332]. The adsorption of adenine was shown to be a function of pH and followed the Langmuir adsorption isotherm. The amount adsorbed and the values of the Langmuir adsorption isotherm on Mt with or without the divalent cations showed that the adsorption trend was mostly determined by the nature of Mt. Jaber et al. [333] investigated the selective adsorption and polymerization of glutamic acid (Glu) and arginine (Arg) on Mt by employing either selective adsorption or wet impregnation. In the first instance, the adsorption selectivity was related to the pH-related speciation of Glu and Arg. At a natural pH, Arg has a positive charge and is consequently significantly exchanged for the interlayer cations in Mt. In contrast, Glu is negatively charged and was adsorbed only in small quantities, most likely on the edges of the Mt particles. In comparison, incipient wetness impregnation forced equal amounts of Glu and Arg to be adsorbed. Following moderate thermal activation, some peptidic condensation between the amino acids was observed, suggesting a selective polymerization that forms heteropeptides (e.g., cyclo(Glu-Arg)) instead of homopeptides.

In addition, the adsorption of nucleic acid bases on different clay surfaces has been evaluated using computer simulations. An ab initio calculation study by Michalkova et al. [334] indicated that uracil adsorption took place perpendicularly to the kaolinite layer surface. In contrast, for Mt, nucleic acid bases were shown to have a tendency to adsorb in a face-to-face orientation relative to the layer surface [334]. Mignon and collaborators investigated the adsorption of nucleobases on the external surfaces of Na^+^-Mt [335], taking into consideration hydration [336], as well as on the acidic external surfaces of Mt [337] (i.e., with H^+^ as the counterion). As Mt is a 2:1 smectite-group clay mineral, nucleobase interactions were only investigated on the basal surfaces of tetrahedral sheets. For Na^+^-Mt, a large set of different complexes, taking into account both parallel and orthogonal orientations relative to the external surfaces, were explored [335]. On the Na^+^-free surface, because dispersion forces formed the nearly complete contribution to the adsorption energy, in the majority of instances, nucleobase adsorption parallel to the silica sheet was determined to be more favorable compared to the orthogonal orientation. On the Na^+^-containing side, various adsorption modes were detected: (a) standard cation–π/ring interactions, (b) cation–π/displaced interactions, and (c) cation–heteroatom interactions. The first two interactions caused parallel adsorption, whereas the last one resulted in orthogonal adsorption, allowing for efficient H-bond formation. Calculated adsorption energies suggested that the cation–π/ring interactions were less favorable compared to the other two interactions because of the smaller influence of electrostatic interactions. On the Na^+^-containing surface, cation–heteroatom interactions were determined to be more favorable compared to the cation–π/displaced interactions for guanine and cytosine, since they formed bidentate coordination (thereby increasing the electrostatic contribution), whereas for adenosine, uracil, and thymine, the cation–π/displaced configurations turned out to be more favorable. In the specific situation of cytosine on the Na^+^-containing surface, hydration effects were also investigated using ab initio molecular dynamics simulations [337]. When cytosine was located close to the external surface, it remained adsorbed on that surface, oriented in a parallel fashion, because it was stabilized through dispersion interactions. In addition, the cytosine O heteroatom formed a cation–heteroatom interaction with Na^+^, staying adsorbed on the Mt surface while partly solvated by water molecules. In addition, the cytosine NH group formed H-bonds with water. In addition, the adsorption of adenine, guanine, and cytosine on acidic Mt surfaces under very dry conditions was explored [337], where both the octahedral and tetrahedral substituted forms were taken into consideration, since they possess different acidic properties. In nearly every complex, a spontaneous transfer of the acidic proton from the Mt surface to the nucleobases was detected, demonstrating the strongly acidic nature of the external Mt surfaces. Adsorption was observed to be more favorable in the octahedrally substituted Mt compared to the tetrahedrally substituted Mt, pointing to higher Brønsted acidity in the octahedrally substituted Mt. Nucleobase adsorption was dominated by H-bond formation (nucleobases functioning as H-bond donors to the Mt surface) and dispersion interactions (of major importance in the case of parallel orientation), in addition to electrostatic interactions between the positively charged nucleobases and the negatively charged Mt surfaces. Glycine adsorption on K^+^-Mt surfaces, in both dry and hydrous conditions, was studied by Escamilla-Roa et al. [338]. Under these very dry conditions, the optimization of glycine located within the interlayer space caused the spontaneous conversion from its canonical form (NH_2_CH_2_COOH) to its zwitterionic form (NH_3_^+^CH2COO^-^). In this optimized structure, the zwitterion’s COO^-^ group interacted with the exchangeable K^+^ interlayer cation, whereas the NH_3_^+^ group interacted with the basal O atoms forming the surface of the tetrahedral sheet. These two interactions accounted for the stabilization of the glycine zwitterion, turning the Mt into a solid solvent. Comparable glycine adsorption calculations were performed, taking into account different hydration levels in the interlayer space of Mt. In every one of these situations, the zwitterion configuration was shown to be the most stable configuration. Remarkably, in every optimized structure for these hydrated Mt structures, the exchangeable K^+^ interlayer cation relocated to the center of the interlayer space, far from the Mt interlayer surfaces, completely solvated by confined water molecules. Lastly, the substitution of the exchangeable K^+^ interlayer cation by glycinium (i.e., the protonated form of glycine, NH_3_^+^CH_2_COOH) was determined to be moderately favorable, which may explain the experimental results indicating that glycine was adsorbed in Mt as the glycinium cation [339].

Serpentines are a group of 1:1 clay minerals (chrysotile, lizardite, and antigorite), commonly formed through the weathering of olivine- and pyroxene-group minerals. As such, serpentines would have formed on the surface of the primitive Earth. Serpentines, though, have a low capacity to adsorb amino acids, although it has been proven that they can adsorb quantifiable amounts of aspartic and glutamic acids [340]. In contrast, allophane can adsorb significant amounts of alanine [341]. The adsorption isotherms exhibited three clear intervals as the (equilibrium) concentration (Ce) of alanine increased: an almost linear increase at low Ce, a flattening to a plateau at intermediate Ce, and a sharp linear increase at high Ce. Likewise, the alanine oligomers did adsorb on allophane, though the adsorbed amount did not change much as a function of the solute molecular weight [342].

Based on the review of the investigations on the role of smectite-group clay minerals in Section 7, it is clear that nearly all of the previous work has been focused on Mt as the main clay mineral. However, in the early history of Earth, the amount of iron-rich smectites and nontronites was probably significantly larger than it is now, considering that they were mainly the weathering products of peridotite and komatiite rocks rich in olivine and pyroxenes. This is even more important in the case of Mars, where nontronite and iron-rich smectites are the most common smectites. That leaves the question of whether all of the results presented here for montmorillonite in terms of adsorption and catalysis are also valid for iron-rich smectites and nontronites, something that remains to be seen. There are indications, as shown in Section 5, that the effect of iron on catalytic reactions can be significant.

### 6.4. Polymerization of Biomolecules

Sugars can be produced from formaldehyde via the formose reaction. Clay minerals can act as catalysts for the self-condensation of formaldehyde. In addition, the sugar oligomers produced are stabilized through adsorption onto Mt surfaces [343,344]. Plate tectonics have been active for most of the geological history of Earth. This means that when organic-rich sediments were transported into a trench near a subduction zone with increased temperatures and pressures compared to the surface, the amount of water in the sediments would be reduced. Therefore, the concentration of organic molecules would increase as a result, supporting polymerization reactions [345]. The synthesis of glycine peptides using Mt as a catalyst under these pressure and temperature conditions (5−100 MPa, 150 °C) resulted in up to 10-mers of glycine [346]. Peptides were formed through the condensation of amino acid adenylates in alkaline solution using Mt [347,348,349]. Without Mt present, polycondensation was the main reaction type, resulting in peptides up to 12 monomeric units in length. The presence of Mt produced a larger number of polymers that additionally had longer carbon skeletons. In the copolymerization reactions of alanyl adenylate and seryl adenylate, peptides with lengths up to about 100 units were observed. In addition, the polymerization reactions on Mt were characterized by the formation of a distinct series of peptides, which was not detected in the reaction without Mt. In a “shock” experiment replicating the impact of meteorites and asteroids on the Earth’s surface, amino acids showed polymerization to oligopeptides (mainly dimers and trimers) [350].

The formation of polynucleotides in the presence of clay as a catalyst was studied by Ferris and colleagues. With 5-phosphorimidazolide of adenine (ImpA) used as the activated RNA monomer, Ferris and Ertem [351] produced oligomers containing 6−14 monomer units with Mt present. Nevertheless, the creation of RNA oligomers is only the first step on the way to forming RNA containing over 40 monomers that are, in theory, necessary for the start of the “RNA world”. Long-chain RNA can be formed by employing the “feeding” method, i.e., through the regular addition of ImpA to the decanucleotide (10-monomer primer) adsorbed to Na-Mt. Polynucleotides with over 50 monomers were produced after 14 feeding cycles, while the main products consisted of 20–40 monomer units [352]. Utilizing activated adenosine-, uridine-, guanosine-, or cytosine-5′-phospho-1-methyladenine, Joshi et al. [353] managed to form 40–50 monomers. Ertem and Ferris [354] studied six binary reactions of 5′-phosphorimidazolides of adenosine, cytidine, guanosine, and uridine using Mt as the catalyst. A 16–51 times higher production of 5′-purine–pyrimidine dimers compared to 5′-pyrimidine–purines was detected. The overall production of the 5′-purine–pyrimidine dimers was between 50 and 70% compared to 1.3–17% for the 5′-pyrimidine–purine dimers. A lower sequence selectivity was found in the homodimers produced. Regioselectivity for the creation of 3′,5′-phosphodiester bonds was observed with Mt present compared to that without Mt. Similar results were reported in another study by Ertem et al. [355]. Ferris et al. [356] and Ferris [357] succeeded in the formation of RNA molecules containing up to 15 monomer units starting with activated nucleotides in reactions performed in aqueous solution catalyzed by Mt. RNA molecules consisting of up to 50 monomer units were produced in multistep reactions. Huang and Ferris [358] were able to produce RNA oligomers larger than 35–40 monomer units in length in a one-step reaction using Mt as a catalyst from unblocked RNA monomers in 1 day at 25 °C in aqueous solution. Miyakawa et al. [359] reported on the oligomerization of activated mononucleotides with Mt as the catalyst to produce RNA oligomers. This reaction was aided by a relatively high concentration (in this case, 1 M NaCl) of salts. In addition, it was observed that the divalent cations did not need to be present for the reaction to proceed. High concentrations of NH_4_^+^ and HCO_3_^-^ together with 0.01 M HPO_4_^2-^ were observed to inhibit the reaction. Joshi et al. [353] observed that nucleotides can form oligomers with lengths up to 50-mers using Mt as a catalyst, but that the activity depended on the nature of the Mt used. It was shown that the activity of the Mt catalyst was related to the negative charge of the Mt layers and the associated number of interlayer cations. When the interlayer cations in the starting Mt were exchanged with Na^+^, the resulting Na^+^-Mt became catalytically inactive since the Na^+^ saturated the interlayer spaces between the Mt layers, thereby preventing the binding of the activated monomers. Reacting Mt with dilute HCl exchanged the interlayer cations on the starting Mt for H^+^. The H^+^-Mt, adjusted to a pH of 6–7, acted as a heterogeneous catalyst for the successful production of RNA oligomers. Catalytically inactive Mt had a higher negative layer charge caused primarily by the naturally present substitution of the tetravalent elements in the tetrahedral sheet and trivalent elements in the octahedral sheet within the Mt layer structure with trivalent and mainly divalent metal ions, respectively. Aldersley and Joshi [360] looked in more detail at the influence of the nature of the Mt used as a catalyst. To date, of the more than 200 different clay minerals investigated as heterogeneous catalysts, only a select few showed excellent catalytic activity. They used less efficient Mt catalysts to determine their performance in the formation of nucleotide dimers where a nucleoside and an activated nucleotide produced only linear products. The two representative Mt samples, Otay and Chambers, while being rather poor catalysts for the direct oligomerization of activated nucleotides, did promote dimer formation. The amounts of various products were always lower compared to those produced using the exceptional catalyst Volclay. These data offer a basis for a better insight into the physical processes involved in the mechanism of Mt-catalyzed reactions and point to the fact that more Mt samples than so far anticipated may have been able to catalyze RNA formation, at least at the dimer level. Joshi et al. [361] examined the reaction of the 5′-phosphorimidazolide of nucleosides with Mt as the catalyst as a model to investigate the prebiotic formation of RNA-type oligomers. The best catalytic activity was found in the pH range between 6 and 9 and salinity of around 1 ± 0.2 M NaCl. After the weathering conditions of primitive Earth that formed catalytically active Mt were changed to simulate the weathered soil conditions expected on Mars, the catalytic activity did not change, with no alteration of the Mt surface layer charge. Furthermore, the catalytic production of oligomers up to tetramers in size was observed when employing just 0.1 mg of Na^+^-Mt, indicating that the potential catalytic activity of a Martian smectitic clay return sample of less than 1 milligram can be studied. Namani et al. [362] investigated a multicomponent environmental system containing amino acids, RNA mononucleotides, and Mt at several Mg^2+^ concentrations. Certain alpha-amino acids, particularly those that were prebiotically most important, behaved as prebiotic coenzymes and further increased the polymerization catalyzed by Mt in a cooperative process to form much longer RNA oligomers. Importantly, and unlike template-directed nonenzymic RNA polymerization by primer extension, the addition of Mg^2+^ was not necessary for polymerization with Mt as the catalyst, in particular when augmented by specific amino acids. Therefore, amino-acid-specific RNA polymerization with Mt as the catalyst is compatible with protocell membranes and could have occurred in a broader range of geochemical environments of different Mg^2+^ concentrations.

### 6.5. Clay Minerals as Potential Information Carriers

The two most important defining functions of current life consist of metabolism (energy transformation) and information storage (DNA and RNA). Clay mineral surfaces have most likely supported the development of metabolism. Did these clay minerals also have some involvement in the initial conservation and reproduction of chemical information? Schneider [363] proposed that intricate dislocation networks present in crystals can, in certain instances, adhere to the principles of living systems, resulting in a crystalline physiology. In addition, the potential natural occurrences of this type of physiology were reviewed, e.g., in terrestrial and extraterrestrial rocks and interplanetary dust. Some 40–50 years ago, Graham Cairns-Smith introduced a similar approach to answer this question in a number of publications [9,284,364], where he proposed that some completely inorganic mineral systems, clay minerals in particular, might be considered the initial living systems, because these minerals would have shown some of the elementary properties of life: their external properties (phenotype) would have been the result of chemical information locked in their internal lattice structures in an orderly fashion (genotype); they might have grown, “reproduced” vegetatively, and at the same time, retained essential information; on top of that, they may have been subjected to a type of natural selection. The genetic transition from the clay mineral to a biochemical system is definitely challenging. Its details are unclear at present; a quite basic problem with this transition is that a specific sequence of information that could have certain survival or reproduction value in the clay mineral system does not have to be similarly adaptive in the biochemical system. Besides some very broad observations that certain crystallographic defects do reproduce during crystal growth [365], one paper in particular studied the crystallization of some saponites in the presence of oxalate [154]. Earlier, Siffert [366] had observed that oxalic acid acted as a catalyst for the crystallization of clay minerals at relatively low temperatures. Clay mineral crystallization is feasible due to the complexation of organic anions with Al, Fe, or Mg cations. Small et al. [367] and Small and Manning [368] proved that oxalate anions destabilized the gel and increasing Al solubility increased 2:1 clay mineral formation. Hartman [369] proposed that the crystallization of Fe- and Mg-rich smectite-group clay minerals, especially saponite, might have been catalyzed by polar organic molecules, e.g., oxalate and amino acids, in the warm lagoons and tidal pools present on the surface of early Earth. Schumann et al. [154] observed that areas of low or high isomorphic substitutions in the saponite layer propagated from one layer to the next one in the stacking. This is an intriguing result, though in this situation, the information is distributed over the surface of the layer. This two-dimensional information is rather different from the one-dimensional information that appears to be more important to the development of biological information, as is locked in nucleic acids in current genetic systems. Zhou et al. [156] studied the crystallization of sauconite as a model for saponite in the presence of succinate. In addition, they showed that seeding with a single sauconite particle caused nucleation at low temperatures, accelerating not only the crystallization process but also the amount of sauconite formed. Comparable to the synthesis of saponite in the presence of oxalate, sauconite crystallization is linked to the capacity of succinate to chelate Al^3+^. Vogels et al. [157] used urea in the synthesis of saponite, as was recently reviewed by Ponce and Kloprogge [155] in this journal. Based on its structure and properties, urea is of interest as a possible starting compound for the formation of nucleobases and related compounds [158,159]. In addition, urea is known to promote phosphorylation reactions [158,159]. Furthermore, it catalyzed the formation of saponite clays in only 20 h at 90 °C [158]. The aluminosilicate gels contain tetrahedrally coordinated Al^3+^, similar to what naturally occurs in, for example, volcanic glass. The decomposition of urea, a molecule thought to participate in various prebiotic organic reactions, prevents the crystallization of brucite, Mg(OH)_2_, during saponite synthesis through the slow formation of ammonia, thus restricting the hydrolysis of Mg^2+^ [160]. Thus, urea potentially played a dual role in the history of early Earth, not only as a simple molecule in various early organic reactions but also as a catalyst in the formation of saponite, which itself can act as a heterogeneous catalyst for other organic reactions.

### 6.6. Encapsulation

There is a single prerequisite for living systems that is essential but has not been discussed yet. The organic molecules must be kept together instead of gradually diluting into the surrounding water or atmosphere. In cells, all of these organic molecules are held together through encapsulation in vesicles surrounded by a semipermeable membrane. Vesicles can be created spontaneously from solutions with dissolved amphiphilic molecules [370]. These amphiphilic molecules, particularly fatty acids, are not that hard to produce under prebiotic conditions. It has been shown that membranes and vesicles can be formed from organic matter obtained from a meteorite [371]. Nevertheless, encapsulation works better if the organic compounds are already highly concentrated. Following Bernal’s original ideas, several minerals have been shown to naturally develop a range of porous structures, from the microporosity of zeolites with dimensions similar to the sizes of small molecules (nanometers) to the micrometer-sized weathering channels (macropores) that can form in feldspars [372], which could have worked well for compartmentalization. Actually, the initial cell wall might well have consisted of an internal mineral surface [373]. The macroporosity present in pumices has also been mentioned [374], while smectite clay minerals with their expandable interlayer spaces may have offered size-selective microporosity for various organic molecules [375]. All of these different types of pores could form a relatively protected system for the self-organization of prebiotic organic molecules, protecting them not only from dilution but also from photochemical degradation caused by the hard UV radiation that reached the surface of the early Earth. After clay minerals have settled on the ocean floor (or dried), they may enclose small spaces. It is possible that these small volumes acted as primitive cells. In addition, when clay minerals are suspended in water, bubbles can form in water or at the surface of water, with the clay particles assembling at the interface between water and air, creating a cell-like spherule [376]. Hanczyc et al. [377] observed that Mt facilitated the assembly of fatty acids into encapsulating vesicles. Additionally, small Mt particles were found in a few of the newly formed vesicles. These encapsulated Mt particles can offer a possible route for the prebiotic encapsulation of catalytically active clay minerals in membrane vesicles. Hanczyc et al. [378] showed that although RNA polymerization on mineral surfaces might be confined to the surface environment presented by Mt, vesicle formation might be increased when different types of surfaces are available. They presented a model where new sheets of amphiphilic molecules can form proximal to an Mt particle surface. Comparable interactions between amphiphilic molecules and mineral surfaces on early Earth could have caused the encapsulation of a variety of catalytically active mineral particles. That the process of forming clay-lined vesicles not only occurs on Earth was shown in a paper by Chatzitheodoridis et al. [379], who observed a biomorphic vesicle in the Nakhla Martian meteorite, consisting of Fe-rich saponitic smectite and amorphous material. The concentric wall encloses an initially hollow void and shows internal layering with contrasting nanotextures but homogeneous chemical composition. It probably acquired its general form from an earlier vesicle in mesostasis glass. During the formation of the spherule, intermittent fluid infiltration episodes caused the formation of saponitic smectite layers around the vesicle walls, transformed pyrrhotite to marcasite, and finally blocked off the vesicle’s wall structure from the rest of the system through the deposition of a layer of iron oxides/hydroxides. Among the probable theories, this specific abiotic setting was regarded as the most realistic explanation for the formation of the vesicle in the Nakhla meteorite, and while persuasive proof for a biotic origin is missing, it is clear that the subsurface of Mars had particular settings where life could potentially develop.

## 7. The Clay Hypothesis and the Origin of Life, a Biochemist’s View

The true complexity of the problems concerning the origin and early evolution of life could not be appreciated until the 1960s, during which we acquired knowledge of the genetic code, the Rosetta stone of molecular biology. It had become clear that replication and mutation were properties of nucleic acids, while catalysis and other functions of the cell were mediated by proteins. This situation could not be primitive; it was this separation between genotype (nucleic acids) and phenotype (proteins) that suggested that simpler systems must have preceded the elegant system of molecular biology. The search for simpler systems leading to the independently reproducible subsystem of our present system was usually thought of in terms of nucleic acids or proteins, but an alternative suggestion based on inorganic crystal growth mechanisms was made by Cairns-Smith [9]. Analogies between the behavior of crystals and organisms have a long history. Troland, for example, observed that replication resembled the laying down of one crystal layer from solution onto a pre-existing layer. However, Cairns-Smith went on to make such ideas much more specific, even proposing that the crystals of choice for the first genetic material might be clay minerals.

Clays, because of their enormous surface area and common occurrence (approximately 50% of sedimentary rocks), had already been suggested as sites where concentration and catalysis could take place. In Bernal’s view, clays would be important in the origin of life, but their role was more passive, as they did not replicate but only catalyzed reactions [283]. Schrödinger [380] had speculated on the nature of the modern genetic material before the role of DNA was understood. He suggested that genes would turn out to be “aperiodic crystals”. As Schrodinger put it, “compared with the aperiodic crystal, they [homogeneous crystals] are rather plain and dull. The difference in structure is one of the same kinds as that between an ordinary wallpaper in which the same pattern is repeated again and again in regular periodicity and a masterpiece of embroidery, say a Raphael tapestry, which shows no dull repetition but an elaborate, coherent, meaningful design by the great master”. In a sense, the clay theory proposed the evolution of a two-dimensional chemical tapestry by natural selection. Cairns-Smith [9] stressed that clays were by no means simple homogeneous crystals but full of irregularities of various sorts. Thus, information might be encoded in the substitutions of one ion (e.g., magnesium) by another ion (e.g., iron) or by dislocations. By analogy with DNA, the distribution of ions in the clay structure might encode information that could replicate, given the right conditions of crystal growth, where, for example, new layers form on pre-existing layers; clays such as montmorillonite are unlike three-dimensional crystals in that any such replicated layer could easily separate from the nucleating layer, allowing new adsorptive and catalytic surfaces to be immediately functional. The two-dimensional character of clay minerals allows them to interact with the aqueous environment more fully than three-dimensional crystals, where the interior is inaccessible.

Here was a suggestion, given in some detail, that made clays not merely catalytic but able to replicate, mutate, and evolve—in other words, the first organisms. However, this generated a new problem. How could a system of replicating clays evolve into the modern cell with its genetic code? This problem was taken up by Hartman, who suggested that the whole of central biochemistry evolved, in conjunction with replicating clays, from (mainly) carbon dioxide, nitrogen, and water [10]. The evolution of metabolism in these proposals contradicted Horowitz’s idea that metabolism had evolved from the outside inwards [381]. Granick’s proposal that “Biosynthesis recapitulates Biopoesis” was revived [382]. Biosynthetic pathways were seen to have evolved from carbon dioxide fixation through the citric acid cycle, culminating in amino acids and nucleotides. The evolution of metabolism was from the inside outwards. Hartman considered iron-rich clays as particular candidates for primitive systems [369]. Hartman further went on to propose an evolutionary pathway leading to the genetic code [383,384]. According to this speculation, the first polypeptides were structural and only later evolved their full catalytic potentiality. In replicating clay systems, complex organizations would evolve by adding to simpler systems, and thus, the evolutionary path could be reconstructed. Thus, we formed the ideas that generated the Glasgow Workshop: Just how central might clays have been to the origin of life on the Earth?

### 7.1. Clay Replication

In 1981, a paper was published by Weiss [286] that put the Clay hypothesis front and center among considerations on the problem of the origin of life. With phyllosilicates as models, the notion of replication, i.e., the spontaneous self-multiplication of an information carrier, can be proven to be a common property of some macromolecular systems. Mistakes in replication and feedback along with environmental effects may result in mutants with higher or lower replication rates, thereby facilitating evolution. Taking these findings into consideration, the question of whether or not chemical evolution resulted directly in the nucleic acid/protein system, i.e., the genetic basis shared among all living systems recognized thus far, has to be answered. It seems possible that as an initial step, a much simpler replicating system had developed: such a system may subsequently have experienced the evolution of replicating systems, producing the final nucleic acid/protein system. The principle of replication and self-multiplication is not restricted to the nucleic acid/protein system; it is a more general property of distinct macromolecular systems. More primitive forms of life or types of protolife, therefore, must also be discussed in the context of the origin of life. Perhaps the question of how nucleic acids and proteins were formed and aptly selected in the course of chemical evolution has been incorrectly phrased. Both might have developed in the course of the evolution of replicating systems. The highly expanding clay minerals are an excellent replicating system model; in the course of an immense number of replications, they might have experienced evolution and selection, forced on the system by the environment, in just the manner expected for the most primitive forms of protolife. The paper had many graphs and diagrams and made the case for the Clay hypothesis. It was well written and was very convincing, but no methods were described.

When the Armin Weiss paper was published, John Lewis and Hyman Hartman immediately thought that a Gordon Conference on the Origin of Life should be organized, so the Gordon Conference on the Origin of Life was begun in 1982 and has flourished since then. Lewis managed the planetary section, and Hartman coordinated the rest, concentrating on Clays and the Origin of Life with an emphasis on the Weiss paper. Hartman invited clay chemists that he knew and hoped that they would critically consider his claims. There was an added bonus for him in that he met Graham Cairns-Smith for the first time at this Gordon Conference. The conference was a great success, as the clay chemists were impressed with Armin Weiss, so Hartman proposed to meet in Glasgow at the University where Cairns-Smith was a lecturer. Having received funding from NASA, he met with other attendees in Glasgow for what was a set of talks that were to be written up for a book that would be published by Cambridge University Press. There were 26 speakers. This resulted in the book: *Clay Minerals and the Origin of Life*, edited by Cairns-Smith and Hartman [385].

The Glasgow meeting was held in 1983. It was thought that all of the chapters would be written by 1984. There were 25 authors of the book, and it was published in 1986, three years after the meeting. Why did this happen? It happened because there was a problem, and that was that the 26th speaker was Armin Weiss. The whole book was designed to culminate with Armin Weiss’ contribution to the book, this time providing the experimental details so that the experiments could be repeated in other laboratories. For two years, the editors asked for his chapter, and each time, another disaster occurred that prevented him from finishing his chapter. Finally, someone went to Munich and checked the references in his 1981 paper and found that they were irrelevant. Arrhenius et al. [287] were forced to send a letter to Angewendte Chemie indicating that there were troubling questions concerning Armin Weiss’s paper. In a paper published in 1981 in Angewandte Chemie, Professor Armin Weiss described observations with potential importance for the origin and early evolution of life [287]. This paper has been widely cited since it claimed experimental proof of clay mineral replication, mutational variability in replicating clay crystals, and specific catalytic effects. The results have been invoked in support of the idea that an inorganic evolution through natural selection preceded the evolution of organic life. The information presented in the 1981 Weiss paper is altogether inadequate for others to repeat the key experiments that are said to be “proof of replication”. A single reference in this section reads: “Unpublished results: published in part in G. Mai, Dissertation, Universitat München 1969; P. Brunner, ibid. 1978; S. Fritz, ibid. 1978”. Arrhenius et al. [287] were unable to find the first and third of these theses, while the second had no information that they deemed to have specific relevance to clay replication, nor were repeated attempts to obtain the experimental protocol from Professor Armin Weiss successful. Armin Weiss, like King Ludwig, had built castles that were based on his imagination, but Weiss’ castles were in his head and on paper. This was to be, in the context of a scientific project, the worst possible scenario: specifically, an experimental paper makes a central claim and then does not publish the methods in detail so that others can repeat the experiment. Hartman was furious that he had invested time and NASA money in what had turned out to be a fraud. The damage was done. Clays were no longer at the center of the discussion. The recovery would be long and painful.

### 7.2. Clay Interactions with Peptides

**Scheme 12:**
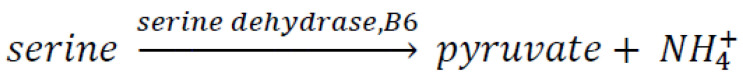
Reaction of serine to form pyruvate using a coenzyme and clay as catalysts.

Hartman met Gerry Soffen in Cambridge, MA, who, at that time, was a project scientist for NASA’s Viking program of Mars landers, the first successful mission to operate unmanned experiments on the Martian surface. As such, he supervised all scientific experiments performed by both landers, managing more than 70 scientists around the USA. Hartman told him about the Clay hypothesis, and he suggested collaborating with a group working at NASA Ames led by Jim Lawless (smectites) and Sherwood Chang (kaolinites). They were working on clays, especially on their interaction with peptides. Hartman joined Jim Lawless’ group and spent three summers there (1978, 1979, and 1980). He spent three summers adding coenzymes, especially pyridoxal phosphate (vitamin B6), to clays. He began with the serine + pyridoxal phosphate reaction. The enzyme is called serine dehydratase (Scheme 12).

**Scheme 13:**
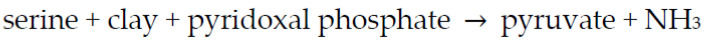
Reaction of serine to form pyruvate using clay and coenzyme as catalysts.

The enzyme is composed of a (protein) apoenzyme + a coenzyme (pyridoxal phosphate). Could the clay substitute for the protein (apoenzyme) (Scheme 13)?

The clay used was Wyoming bentonite—a montmorillonitic clay that was developed from the alteration of volcanic ash in seawater. The pyridoxal phosphate was added to the Mt in a buffered solution, and the reaction was monitored at various temperatures. The experiment worked beautifully—the Mt did substitute for the protein. Then, Hartman ran a control, which entailed the removal of the Mt and addition of the supernatant to serine and pyridoxal phosphate, and it worked as well. He learned that E.E. Snell, a biochemist, had found that serine underwent this reaction using pyridoxal phosphate and metal ions such as Cu^2+^ and Al^3+^. Serine underwent rapid transamination by pyridoxal phosphate in an Al^3+^- or Cu^2+^-catalyzed reaction. There was no need for clay. The bentonite was not a pure compound, and there were ions in the supernatant not attached to the clay. While Hartman was conducting these experiments, Max Mortland visited his laboratory. He was intrigued by these experiments. Mortland decided to focus on enzyme transaminase with the apoenzyme as a protein and pyridoxal phosphate as a coenzyme. Hartman decided not to publish this paper, as Snell had already published his results [386].

**Scheme 14:**
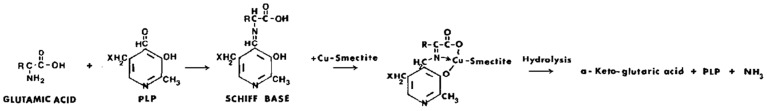
Reaction of glutamic acid and pyridoxal phosphate to form α-ketoglutaric acid with Cu^2+^-smectite as the catalyst.

Mortland [387] showed that glutamic acid was selectively deaminated through a mixture of pyridoxal phosphate (PLP) and Cu^2+^-smectite. Ammonia was formed together with α-ketoglutaric acid (Scheme 14).

This reaction was far more active with Cu^2+^-smectite compared to when the Cu^2+^ cation was simply in solution. Likewise, Cu^2+^-kaolinite, vermiculite, and faujasites X and Y showed some activity, though at a significantly lower rate, indicating that their activity was probably confined to external surfaces only, whereas that of the smectites involved catalysis in the interlayer spaces. Following the addition of a strong ligand (o-phenanthroline) to the reaction, the reaction rate dropped, clearly indicating that coordination of the reacting species with Cu^2+^ on the smectite was essential. The suggested reaction pathway consists first of the formation of a Schiff base between the amino acid and pyridoxal phosphate, followed by complexation with Cu^2+^ at the smectite surface. Next, hydrolysis of the complex to NH_3_ and the α-keto acid occurs, or transamination takes place to form pyridoxamine phosphate, which is subsequently deaminated by Cu^2+^-smectite. The pyridoxal phosphate-Cu^2+^-smectite behaves like a pseudo enzyme, with the clay structure replacing the apoenzyme. Mortland thanked Drs. H. Hartman of the Massachusetts Institute of Technology and J. G. Lawless of the Ames Research Center (NASA), Moffett Field, CA, for sharing their results on pyridoxal phosphate–clay interactions with serine. The reaction mechanisms in both the glutamic and serine cases with pyridoxal phosphate and Cu^2+^ were identical. Mortland’s paper then stimulated others to explore the catalytic properties of clay minerals with coenzymes and metal ions.

**Scheme 15:**
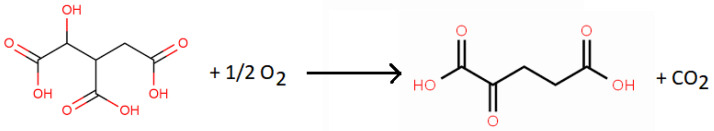
Reaction of isocitric acid conversion to α-ketoglutaric acid with Na^+^-montmorillonite as the catalyst.

The catalytic activity of smectites in numerous chemical reactions led various researchers to study their possible involvement in the origin of life [385,388,389]. Smectite-catalyzed amino acid polymerization has been shown. These initial results led researchers to think about clays playing the role of enzymes in biological systems. Several researchers, in particular, Mortland [387], Boyd and Mortland [390,391], Siffert and Naidja [392], and Naidja and Siffert [393], studied the catalytic activity of smectites in some simple biochemical reactions. The research by Naidja and Siffert [393] was aimed at learning more about the role of clay minerals as catalysts in certain biochemical reactions. They looked at the oxidative decarboxylation reaction of isocitric acid, which forms the third step of the Krebs cycle. The isocitric acid oxidative decarboxylation reaction was studied with and without homoionic Na^+^, Mn^2+^-, and Cu^2+^-Mt present. The catalytic activity of the Mt was a function of the nature of the interlayer exchangeable cation. Isocitric acid was converted to α-ketoglutaric acid in the presence of Na^+^-Mt, where Na^+^ does not produce a complex with the isocitrate anion (Scheme 15).

Instead, with Na^+^-Mt, the isocitrate anion was oxidized and converted to the α-ketoglutarate anion. The interlayer exchangeable Na^+^ ions first permitted the intercalation of isocitrate in the interlayer spaces, which did not result in the formation of complexes with isocitrate. Divalent cations (Mn^2+^ and Cu^2+^) were removed from the interlayer spaces, which resulted in the formation of “external complexes” with the isocitrate anions. However, the reaction rate was far lower than with the enzymatic system (isocitrate dehydrogenase enzyme and nicotinamide adenine dinucleotide phosphate coenzyme). The outstanding transition metal involved in the origin of life is iron, especially Fe^2+^ and Fe^3+^ in the iron clays found on Mars and carbonaceous chondrites. This was clear to Hartman when he convinced Richard Frankel at the Magnet Laboratory (MIT) to investigate the coenzyme flavomononucleotide (FMN) and Fe-clay.

Mortland et al. [394] observed that the adsorption isotherms and UV–visible and Mossbauer spectroscopic data suggested specific interactions between FMN and Fe^3+^-smectite. The highest adsorbed FMN amount was determined to be 0.3 mmole/g Fe^3+^-smectite, yielding a 1:1 molar proportion of Fe^3+^ to FMN. This pointed to the presence of a Fe^3+^–FMN complex on the smectite surface. Other homoionic smectites (Cu^2+^, Zn^2+^, and Ca^2+^) showed less adsorption and less obvious specific interaction. It is likely that the observed interaction takes place between the Fe^3+^ on the smectite and the phosphate group on the sugar moiety of FMN. Since this study, the emphasis has been on transition-state metals rather than coenzymes. However, the behavior of the transition-metal ions in and on the clay lattice is significantly different from their behavior in solution.

### 7.3. Clay Minerals on Carbonaceous Chondrites

In 1980, Hartman went to MIT, where he joined John Lewis in the Earth Atmosphere and Planetary Sciences. He had received a grant from NASA to work in the field of Clays and the Origin of Life and began working with John Lewis on carbonaceous chondrites. Becker and Epstein [239] performed solvent extractions with CCl_4_ and CH_3_OH on the carbonaceous chondrites Murray, Murchison, Orgueil, and Renazzo. Around 2–10% of the total carbon in these meteorites could be extracted using normal methods, mostly in CH_3_OH. The extracts from Renazzo exhibited isotopic ratios indicating that they consisted primarily of terrestrial organic matter, with smaller amounts from indigenous organics. The CH_3_OH-soluble organic matter from Murchison and both untreated and HF-treated Murray showed δ^13^C values between approximately +5 and +10‰ and δ^13^N values between approximately +90 and +100‰, both of which are substantially higher than those determined for the bulk meteorites. Likewise, the Orgueil CH_3_OH extract exhibited a δ^15^N value considerably higher than that of the residual organic matter. δD values between +300 and +500‰ were observed for the CH_3_OH-soluble organic matter. These results for C, H, and N isotopes make it extremely doubtful that the CH_3_OH-soluble components were derived from, or simply related to, the insoluble organic polymer present in these meteorites. Organic matter soluble in CCl_4_ contained almost no N and had lower δ^13^C and δD values compared to the CH_3_OH-soluble compounds. Either large isotopic fractionations for C and H occurred between different soluble organic compounds, or the less polar compounds were in part of terrestrial origin. They concluded that the soluble organic material (amino acids, etc.) was probably introduced to the meteorites, along with high-δ^13^C carbonates, during a hydrothermal event that produced hydrous silicates in CI1 and CM2 meteorites. Hartman et al. [249] indicated that organic compounds found in carbonaceous chondrites could be divided into three different fractions. The first fraction, which is insoluble in chloroform and methanol, has a portion that is of interstellar origin. The remaining two fractions, which are chloroform-soluble hydrocarbons and methanol-soluble polar organics, were assumed to have been formed on a planetoid body. They suggested that the polar organic compounds, i.e., amino acids, were formed near the surface of the chondrite through the radiolysis of hydrocarbons and ammonium carbonate in a liquid water environment. Certain hydrocarbons could have been produced through a Fischer–Tropsch mechanism inside the chondrite body. The ferrous ion was thought to function as a protection against reverse reactions. The concurrent formation of Fe-rich clays and polar organics may be an indication of events associated with the origin of life on Earth. During the outgassing of the early Earth, events similar to those observed in the carbonaceous chondrites must also have taken place. In particular, in recent years, a new model for the origin of life has been proposed. It emphasizes the role of liquid water, Fe-rich clays, and ultraviolet light in the production of biologically significant molecules. Thus, although the radiation involved is not ultraviolet, carbonaceous chondrites may provide invaluable indications of the early steps that led to life on pre-Archean Earth.

In the summers that Hartman spent at NASA Ames, discussions were centered on how Viking was a failed mission, so he told the exobiology division what he thought about Mars and the Viking Mission, which was that they were looking only for organic molecules and completely ignoring the clays on Mars. They had set the laboratories of Sherwood Chang and Jim Lawless to study clays and paid no attention to what was happening in those laboratories. His ideas were published as an abstract at a meeting on Exobiology and Future Mars Missions [395]. To detect life in the Martian soil, the two Viking landers contained tests that were developed to search for respiration and photosynthesis. These two experiments (labeled release, LR, and pyrolytic release, PR) searching for life in the Martian soils provided positive results. However, no organic molecules were found in the soils of Mars. The explanation provided was that the inorganic compounds present in the Martian soil caused these results. The inorganic composition of the Martian soil was best modeled with a mixture consisting of 60–80% clay, iron oxide, and quartz, together with soluble salts such as halite (NaCl). The minerals most effective in replicating the PR and LR tests were Fe-rich clays. One theory considers clays to be the first organisms able to replicate, mutate, and catalyze and, therefore, to evolve. Clays were formed as a result of the weathering of rocks by liquid water. The distribution of ions, e.g., of Al, Mg, and Fe, was thought to act in a manner similar to the sequence of bases in DNA. The information was stored in the distribution of these ions in the octahedral and tetrahedral sites in the clay layers, and they might, similar to RNA and DNA, replicate. When the clays replicated, each clay layer would act as a template for the next layer. The ion substitutions in one clay layer would result in a complementary or similar pattern in the next clay layer formed on its surface. It was hypothesized that on the surface of replicating Fe-rich clays, CO_2_ might react under light to form organic acids, e.g., formic or oxalic acid. If Mars had liquid water during a warm period in its past, clay formation would have been abundant, something that has since been proven to be the case. These clays would have been able to replicate and evolve until the liquid water disappeared as a result of the cooling of Mars. His suggestion was that they should study the Fe-rich clays of Mars. Since that time, Fe-rich clays have become central to the missions to Mars and to the origin of life on Mars.

### 7.4. Clay Surface Analysis with Atomic Force Microscopy

In 1988, Hartman moved back to the University of California, Berkeley, where he met Garrison Sposito in the Soil Science Department. They began to study smectites using an atomic force microscope (AFM). The only path that he could envision at the time was to explore new experimental techniques such as Mossbauer spectroscopy and atomic force microscopy. For this purpose, Hartman et al. [396] purified Mt samples of Crook County and Silver Hill Illite and prepared the Na forms, which were subsequently studied under 80% relative humidity with an atomic force microscope. Direct imaging distinctly showed the hexagonal rings of basal oxygen atoms of the SiO_4_ tetrahedra in the tetrahedral sheet in Mt. AFM might provide useful information at the molecular-scale resolution of clay mineral surfaces such as Mt containing adsorbed organic molecules. This period was followed by several years in which he was not involved in any research related to clay minerals and the origin of life.

### 7.5. Low-Temperature Clay Synthesis and Replication

Hartman returned to the clay world and decided to replicate clay minerals. It began when he spent the summer of 2005 with Dennis Eberl, who was with the USGS, Boulder, Colorado. They were motivated to explore the possibility that oxalic acid could catalyze the formation of clays from a gel. This was again inspired by the work of Becker and Epstein [239], who showed the coupling of clay formation on carbonaceous chondrites with dicarboxylic acids, amino acids, and other water-soluble organic chemicals. This experiment was run, and the results were analyzed by a group of clay chemists led by Hojatollah Vali (McGill University). The research was carried out by Dirk Schumann, a graduate student, which resulted in his PhD thesis and a paper in *Astrobiology* [154]. The possible role of clay minerals in the abiotic origin of life is the topic of ongoing debate that started several decades ago. The main focus is on the clay minerals detected in a class of meteorites called carbonaceous chondrites. These clay minerals were formed through aqueous alteration of anhydrous minerals, e.g., olivine and orthopyroxene, that are frequently found in the chondrules. Furthermore, a strong correlation exists between the existence of these clay minerals and the occurrence of polar organic molecules. Laboratory experiments have proven that at low temperature and ambient pressure, polar organic molecules, e.g., the oxalate ion observed in meteorites, are able to catalyze the formation of clay minerals. In this particular investigation, it was proven that oxalate was a strong catalyst in the formation of saponite, an Al- and Mg-rich trioctahedral clay mineral, from a silicate gel at a temperature of only 60 °C and ambient pressure. High-resolution transmission electron microscopy (TEM) of the synthetic saponite intercalated with octadecylammonium cations showed the presence of 2:1 layer structures with variable negative layer charge. The formation of these differently charged 2:1 layers within the saponite particles most likely took place independently. If polar organic molecules, such as oxalate, are able to catalyze the crystallization of clay minerals, which then can promote the formation of clay microenvironments and offer a large number of adsorption sites on the clay surfaces for other organic molecules existing in the solution, the interaction between the adsorbed molecules could result in polymerization, resulting in more complex organic molecules, such as RNA from nucleotides on early Earth. The major findings of this paper were twofold: (1) Oxalic acid was a catalyst of clay formation. This was suggested by B. Siffert in a chapter in *Clay Minerals and the Origin of Life* entitled “The role of Organic Complexing Agents” [397]. He stated, “A number of successful attempts to synthesize aluminum phyllosilicates at ambient temperature have been reported. In most cases organic aluminum complexes were used”. (2) The crystallization of these differently charged saponites most likely occurred independently. The fact that saponites with variable charge formed from the same gel has consequences for our interpretation of how life originated, as these 2:1 clay minerals most likely replicate via a process of template-catalyzed polymerization and transmit the charge distribution from one layer to the next layer. The reason that the second finding was so interesting was that Hartman had discussed his publication on the formation of clay minerals in soils with Isaac Barshad in the Soil Department at the University of California, Berkeley. He pointed out that his experience with clay formation in soils was that where conditions for clay formation were the same, the clays formed a diverse assemblage of different clays. The reason for his observation was that clay formation is catalyzed by seed clays [398].

The synthesis of clays in the years 2005–2006 took 3 months at 60 °C. This was not going to allow a lot of experiments to be performed, so Hartman searched for a simpler and shorter synthesis at temperatures lower than 100 °C. Then came the reason that he became the editor for Clays and the Origin of Life in the journal *Life*. He came across the paper by Kloprogge et al. [130] on the synthesis of smectite clay minerals, and on page 533, he read the following paragraph:

“Following a new approach, Vogels et al. (1995) synthesized saponites at 90 °C from a Si-Al gel and a solution containing urea and an M^2+^-nitrate [M^2+^ = Zn, Mg, Ni, Co] in only a few (5–20) h. Precipitation from homogeneous solution was induced by the slow hydrolysis of the urea, which resulted in a homogeneous release of hydroxyl ions. The octahedral divalent metal had a large influence on the characteristics of the saponite product, such as stacking order, surface area, and pore volume. With Mg the nucleation and growth were rather slow compared to saponites containing Co or Zn. After 20 h with Mg some gel was still present and even after 2 d almost no stacking was observed by transmission electron microscopy (TEM) in agreement with the absence of the (001) reflection in the XRD pattern. The results with different divalent cations indicated that stacking increased in the order Ni = Mg < Co < Zn. Both the surface area and pore volume increased as Zn < Co < Ni < Mg. The A1 distribution over the tetrahedral, octahedral, and interlayer sites was influenced by increasing the initial Si/A1 ratio in the starting gel from 5.7 to 39. Adjusting both the octahedral-sheet and tetrahedral-sheet composition by the choice of divalent metals or combinations of metals and the Si/A1 ratio offers the possibility to control properties like surface area, pore volume, and acidity” [399]. Hartman immediately began to synthesize Zn-clays using the method of Vogels et al. [399] in Roger Summon’s laboratory at MIT, and it worked. Hartman modified it by adding oxalic acid and NaOH, and this worked as well. He then contacted Marcelo Guzman at the University of Kentucky, and they began a collaboration with his graduate student Ruixin Zhou. The synthetic Zn-clays were characterized by Chris Matocha and Hojatollah Vali and his group (McGill University). The role of primordial metabolic networks, e.g., the reverse tricarboxylic acid (rTCA) cycle, and the coevolution of clay mineral catalysts in the origin of life is still not properly understood. Although prebiotic reactions from the rTCA cycle have been achieved through photochemistry on semiconductor minerals [400], the formation of clay minerals has been shown to be catalyzed by oxalate at low temperature and ambient pressure [154]. Zhou et al. [156] reported on the succinate-catalyzed crystallization of sauconite as a model for clay minerals using a photoproduced intermediate from central metabolism as an example. In addition, they showed that seeding induced nucleation at low temperatures, speeding up the crystallization process. Their results indicate that the coevolution of clay minerals and early metabolites on the Earth’s surface might have been accelerated by sunlight-induced photochemistry, which played a major role in the intricate interactions between rock surfaces and life at a geological timescale. The most important finding is that seeding increased crystallization. The catalytic power of the synthesized clay was validated by replicating the synthesis at 70 °C after adding a single (macroscopic) sauconite particle obtained from the 90 °C synthesized product to the starting gel. The fact that a single sauconite particle acted as a seed crystal to produce a larger amount of sauconite provides an excellent example of the self-catalytic power of clay minerals with respect to their nucleation and crystallization. The surface of the seed particle is thought to enable heterogeneous nucleation at lower temperature via a reduction in the activation energy for crystallization. The detected acceleration in crystallization by seeding does not merely depend on random events but is the result of clay surface interactions with chemical species present in the starting gel at 70 °C. The seed particle interaction with soluble free and complexed ions moving freely in the gel creates intermolecular forces that are required to produce the crystal lattice. The addition of a seed particle to the gel offers a pathway to direct a process that is then independent of random interactions.

## 8. Conclusions

Over the past decades, there has been intense debate about the origin of life, and extensive research has provided some answers. It has been shown that simple organic molecules could have formed from water, methane, ammonia, and hydrogen exposed to light. In addition, it is possible that a variety of organic molecules reached Earth through meteorite impacts. One of the outstanding questions is how the variety of simple as well as rather complex organic molecules came to be on meteorites, asteroids, and comets. Often, these organic molecules have been observed to be associated with clay minerals. Clay minerals have been observed on all of these celestial objects, as well as on Earth and Mars. In recent years significant progress has been made in the identification of clay minerals [35,119,120,121,122] and organic molecules [222,223,228,230] in the Gale crater on Mars by the Curiosity rover showing that about 3.5 billion year old fluvio-lacustrine mudstones in Gale crater consist of nearly 30 wt % of clay minerals [34,35,120]. Though perennial lake mudstones are typified by Fe-saponite, Bristow et al. [121] observed that stratigraphic in-tervals associated with episodic lake drying comprise Al-rich, Fe3+-bearing dioctahedral smectite, with minor amounts of ferripyrophyllite, thought to be wind-blown detritus. Smectite interlayers in the John Klein sample from Yellowknife Bay collapsed somewhere after clay mineral formation and before the analysis to a basal spacing of 10 Å, but mostly stayed expanded in the Cumberland sample with a basal spacing of about 13.2–13.5 Å. It is uncertain if the 10 Å basal spacing for most phyllosilicates is related to smectites that collapsed due to dehydration, or if these samples contained partially illitized clay miner-als with non-expandable interlayer spaces due to the incorporation of K+ in the ditrigonal cavities, as would be the case if glauconitic clays were present. Analysis by Losa-Adams et al. [401] showed that these clay minerals are structurally and compositionally related to glauconitic clays that form a sensitive proxy of calm conditions in liquid bodies for long periods of time. This points to long periods of very low sedimentation in an ancient brackish lake on Mars, characteristic of an aqueous system with slow evaporation at low temperatures. Clay minerals on Earth are essential for protecting and preserving organics. On Mars, clay minerals are important for exploration as they are thought to be an indica-tor for potential habitable environments. CheMin data have shown that ancient flu-vio-lacustrine rocks in Gale crater contain up to about 35 wt.% of clay minerals. They are important markers of past fluid–rock interactions, and variation in the structure and composition of these clay minerals in Gale crater indicate variations in past aqueous en-vironments that might have been suitable for microbial life.

It is a major step from these rather simple organic molecules to the complex systems that we now know as living cells containing important molecules for replication, such as RNA and DNA. How did life on Earth evolve from just simple chemical compounds to these complex systems? Catalysis on mineral surfaces, particularly clay minerals of the smectite group, may bridge this gap. It is well known from industrial applications that smectite-group clay minerals such as montmorillonite can catalyze a large variety of organic reactions. It is highly likely that similar clay-catalyzed reactions could have happened on the surface of early Earth. More research on this aspect is necessary. On early Earth and on Mars, montmorillonite was probably not the most important clay mineral; instead, Fe-rich smectites and nontronites were dominant. Thus far, relatively little research has been conducted on the catalytic properties of these iron-containing smectites, and it is not clear if the results obtained for montmorillonite can be directly translated to nontronite and Fe-rich smectites. In addition, clay replication with organic molecules, such as oxalate and urea, as catalysts has only been shown for saponite and sauconite but not for montmorillonite and nontronite [154,155,156,157]. Only a single paper has shown the effect of seeding on the formation of these clay minerals [156], and this aspect needs to be further investigated. An interesting paper in that respect was recently published by Besselink et al. [161]. It would be very interesting to replicate their experiments but with seeding.

A major unanswered problem is the fact that most amino acids found in living systems are typically L-enantiomers, but under abiotic conditions, both D- and L-enantiomers would have formed in equal amounts. How did the differentiation between the two occur? Glavin and Dworkin [248] observed that the large L-enantiomeric excesses in isovaline, which have previously been detected in Murchison, were also present on the CI meteorite Orgueil. This may indicate that a wide range of carbonaceous chondrites could have delivered meteoritic amino acids contributing to the origin of homochirality on Earth and possibly elsewhere. Since aqueous alteration on these meteorite parent bodies (i.e., clay formation) probably played a significant role in the increase in L-isovaline excesses, it is quite possible that these L-amino acid excesses increased even more in the aqueous environment on early Earth. Chiral mineral surfaces have been postulated as a plausible source of symmetry breaking at the early stages of life on Earth. The edge surface of Mt may show some structural chirality due to the presence of structural defects. Siffert and Naidja [304] observed that Mt exhibited stereoselectivity in the adsorption and deamination of aspartic and glutamic acids. The chirality of clay minerals needs further study. A possible test could be based on the synthesis of a clay mineral in which the chelating agents are optical isomers of, e.g., aspartic acid. The experiment would be to synthesize it with either D- or L-aspartic acid as chelators. The next step would be to test whether the synthetic clay would recognize (by adsorption) the optical isomer of aspartate involved in their synthesis. In conclusion, it is our view that the most promising theory to explain the missing steps in the origin of life is the interaction of active sites on clay mineral surfaces with simple organic molecules. These active sites allowed for the adsorption and protection of organic molecules, catalytic reactions to form more complex organic molecules, and subsequent polymerization. Significantly more research needs to be conducted to further our understanding of all these steps.

## Figures and Tables

**Table 1:** Clay minerals mentioned in this article, clay mineral type, and ideal chemical composition.

Clay Mineral	Type	Chemical Composition
Allophane	Short-range-order clay	(Al_2_O_3_)(SiO_2_)1.3–2·2.5–3H_2_O
Kaolinite	1:1 clays, dioctahedral	Al_2_Si_2_O_5_(OH)_4_
Lizardite	1:1 serpentine, trioctahedral	Mg_3_Si_2_O_5_(OH)_4_
Antigorite	1:1 serpentine, trioctahedral	Mg_3_Si_2_O_5_(OH)_4_
Chrysotile	1:1 serpentine, trioctahedral	Mg_3_Si_2_O_5_(OH)_4_
Talc	2:1 clay, trioctahedral	Mg3Si4O10(OH)2
Montmorillonite	2:1 clays, dioctahedral	(M^+^_y_.nH_2_O)(Al^3+^_2-y_Mg^2+^_y_)Si^4+^_4_O_10_(OH)_2_
Beidellite	2:1 clays, dioctahedral	(M^+^_x_.nH_2_O)Al^3+^_2_(Si^4+^_4-x_Al^3+^_x_)O_10_(OH)_2_
Nontronite	2:1 clays, dioctahedral	(M^+^_x_.nH_2_O)Fe^3+^_2_(Si^4+^_4-x_Al^3+^_x_)O_10_(OH)_2_
Hectorite	2:1 clays, trioctahedral	(M^+^_y_.nH_2_O)(Mg^2+^_3-y_Li^+^_y_)(Si^4+^_4_O_10_(OH)_2_
Saponite	2:1 clays, trioctahedral	(M^+^_x_.nH_2_O)Mg^2+^_3_(Si^4+^_4-x_Al^3+^x)O_10_(OH)_2_
Sauconite	2:1 clays, trioctahedral	(M^+^_x_.nH_2_O)Zn^2+^_3_(Si^4+^_4-x_Al^3+^x)O_10_(OH)_2_
Stevensite	2:1 clays, trioctahedral	(Ca,Na)_x_Mg_3-x_(Si_4_O_10_)(OH)_2_
Vermiculite	2:1 clays, trioctahedral	Mg_0_._7_(Mg,Fe,Al)_6_(Si,Al)_8_O_20_(OH)_4_.8H_2_O
Corrensite	1:1 regular interstratified ^1^	(Mg,Fe)_9_((Si,Al)_8_O_20_)(OH)_10_·nH_2_O
Chlorite	2:1 clays, interlayer octahedral sheet	([Mg^2+^Fe^2+^]_5_Al^3+^)(Si^4+^_3_Al_3_)O_10_(OH)_8_

M^+^ is an exchangeable interlayer cation, such as K^+^ or Na^+^. ^1^ Corrensite is a 1:1 regular interstratification of a trioctahedral chlorite with either a trioctahedral vermiculite (low layer charge corrensite) or a trioctahedral smectite (high layer charge corrensite).

## Data Availability

Not applicable.
